# An Extensive Alien Plant Inventory from the Inhabited Areas of Galapagos

**DOI:** 10.1371/journal.pone.0010276

**Published:** 2010-04-21

**Authors:** Anne Guézou, Mandy Trueman, Christopher Evan Buddenhagen, Susana Chamorro, Ana Mireya Guerrero, Paola Pozo, Rachel Atkinson

**Affiliations:** 1 Charles Darwin Foundation, Puerto Ayora, Galapagos, Ecuador; 2 University of Western Australia, Crawley, Western Australia, Australia; 3 SWCA Environmental Consultants, Honolulu, Hawaii, United States of America; 4 Instituto Mediterráneo de Estudios Avanzados, Islas Baleares, España; Institut Mediterrani d'Estudis Avançats (CSIC/UIB), Spain

## Abstract

**Background:**

Plant invasions are causing habitat degradation in Galapagos. Problems are concentrated on the four inhabited islands. Plants introduced to rural areas in the humid highlands and urban areas on the arid coast act as foci for invasion of the surrounding Galapagos National Park.

**Methodology/Principal Findings:**

Here we present results of the most comprehensive inventory to date of alien vascular plants in the inhabited areas of Galapagos. The survey was conducted between 2002 and 2007, in 6031 properties (97% of the total) on Floreana, Isabela, San Cristobal and Santa Cruz Islands. In total 754 alien vascular plant taxa were recorded, representing 468 genera in 123 families. Dicotyledons represented 554 taxa, monocotyledons 183, there were 7 gymnosperms and 10 pteridophytes. Almost half (363) of the taxa were herbaceous. The most represented families were Fabaceae (sensu lato), Asteraceae and Poaceae. The three most recorded species in the humid rural areas were *Psidium guajava*, *Passiflora edulis* and *Bryophyllum pinnatum*, and in the dry urban areas, *Aloe vera*, *Portulaca oleracea* and *Carica papaya*. In total, 264 (35%) taxa were recorded as naturalized. The most common use for taxa was ornamental (52%).

**Conclusions/Significance:**

This extensive survey has increased the known alien vascular flora of Galapagos by 257 species, giving a ratio of alien to native taxa of 1.57∶1. It provides a crucial baseline for plant invasion management in the archipelago and contributes data for meta analyses of invasion processes worldwide. A repeat of the survey in the future would act as an effective early detection tool to help avoid further invasion of the Galapagos National Park.

## Introduction

When plants are introduced to new environments, some of them will naturalize, and of these some will become invasive [1]. The resultant plant invasions can alter ecosystem and physical processes, reducing the abundance or survival of native species [2]. Recently, Caujapé-Castells et al [3] identified invasive plants as an important threat to endemic plants on oceanic islands. In Galapagos, several species have been recognized as having detrimental effects on native habitats, or transforming the composition and structure of native plant communities [4]–[6]. Fortunately however, in contrast with other tropical high-island archipelagos, Galapagos is still considered to be relatively pristine, with an estimated 95% of its pre-human biodiversity remaining [7].

Within the archipelago, alien plants are found primarily on the four inhabited islands which contain demarcated agricultural and urban areas, and invasions are particularly problematic in the wetter highland regions [8]–[10]. In recognition of the growing problem of alien species, and the need for more comprehensive information and targeted action, a six year, multi-partner program entitled “Control of Invasive Species in the Galapagos Archipelago” was initiated. Between 2001 and 2007, this ambitious and holistic project set out to achieve several interlinked objectives. This included an exhaustive baseline of alien plant species from the inhabited areas of the four populated islands.

Here we present final results of the most extensive inventory to date of alien plants in the inhabited areas of Galapagos. We provide the complete species list, including general species characteristics, and compare this list with the archipelago's native flora and alien floras of other oceanic archipelagos. We discuss the importance of this exhaustive baseline in informing current invasive plant management within the archipelago; concentrating on its use as an early detection tool, in allowing the identification of future introductions and monitoring trends in invasive species spread. We also discuss the benefits and drawbacks of the inventory survey method. Our definition of naturalized species includes both casual and naturalized plants defined by Pyšek et al. [11] as “alien plants that may flourish and even reproduce occasionally outside cultivation, but that eventually die out”, and as “alien plants that sustain self-replacing populations for at least 10 years without direct intervention by people” respectively. Likewise we follow the definition of Pyšek et al. for invasive plants: “naturalized plants that produce reproductive offspring, often in very large number, at considerable distances from the parent plants, and thus have the potential to spread over a large area”.

Mack et al [2] have acknowledged both the importance and difficulty of identifying future invaders and preventing their dispersal and establishment. It is with this in mind that we present this study. We hope the results can aid the identification of potential invaders and allow for a timely response to prevent future establishment and spread into native ecosystems.

## Methods

### Study area

**Figure 1:**
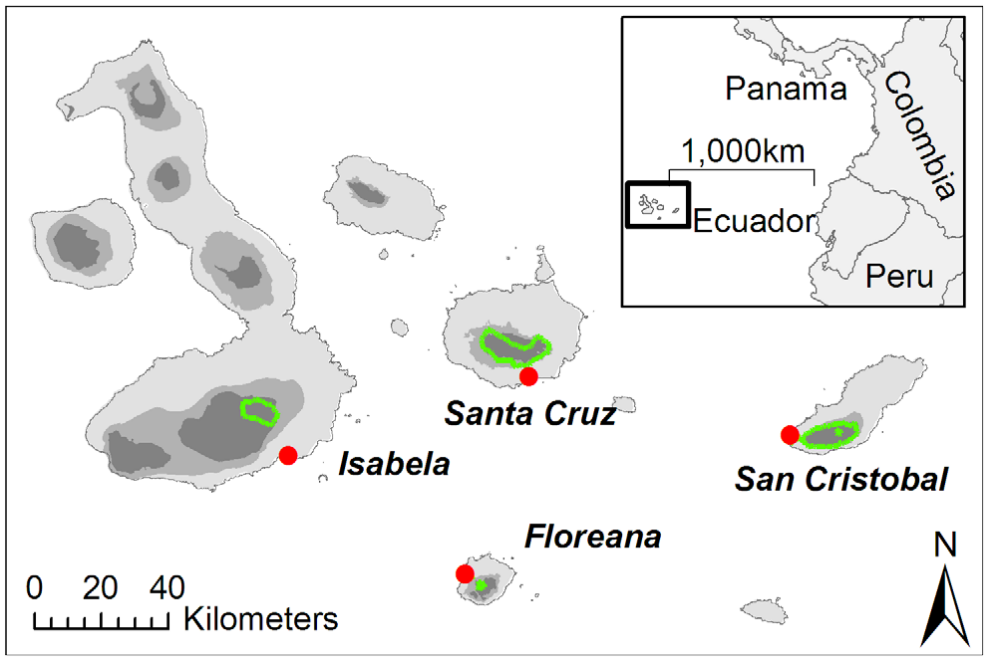
Location map of Galapagos, showing the broad climatic zones (light: arid, medium: transition, dark: humid). The four permanently inhabited islands are labelled, and the inhabited areas shown within green lines (rural) and red points (urban).

A province of Ecuador, Galapagos is an archipelago in the Pacific Ocean, lying on the equator, approximately 1,000 km west of the South-American mainland (Figure 1) (1°40′N–1°36′S, 89°16′–92°01′W) [12]. It consists of 123 islands of volcanic origin, rising from two metres to nearly 1,700m above sea level. The Galapagos Islands were first formally settled in 1832, but had been visited by pirates, whalers and sealers since their discovery in 1535 [13]. Subsistence agriculture began in the early 1800s and remained together with fishing as the major economic activity, until the 1960's. Four islands (Floreana, Isabela, San Cristobal and Santa Cruz) are now permanently inhabited within specifically delimited areas (a fifth inhabited island, Baltra, with a military base and civil airport, is not considered in this study). Each of the four settled islands has a port town in the dry coastal zone, where most of the population live, and a rural area in the humid highlands used mainly for agriculture (Figure 1). In 1950, prior to the formation of the National Park, there were only an estimated 1346 residents. In total, the inhabited areas cover about 3% of the land area; the remaining 97% comprises the Galapagos National Park, created in 1959. Access to protected land is largely limited to guided tours in specific areas, and scientific and conservation management efforts. The archipelago's human population started to increase rapidly from the 1970's onwards, in parallel with the growth of tourism [14], and in 2007, the population of the archipelago was estimated at 19,184 [15]. On Santa Cruz and San Cristobal, the most populous islands, the population has been increasing exponentially over the past 30 years [16].

### Data collection

#### Sampling methodology

Species inventories were carried out between 2002 and 2007. The Santa Cruz urban area (Puerto Ayora) was surveyed between August 2002 and February 2004, and the rural area in July 2004. The Isabela urban area (Puerto Villamil) was surveyed between October 2004 and September 2005, and the rural area between October 2004 and November 2005. The San Cristobal urban area (Puerto Baquerizo Moreno) was surveyed between May 2006 and April 2007, and the rural area between June 2006 and November 2007. Floreana urban (Puerto Velasco Ibarra) and rural areas were surveyed in August 2006.

In the rural areas of Floreana, Isabela and San Cristobal, every property was visited and for each, a full inventory of all alien plant species detected was compiled. In the rural area of Santa Cruz, only 10 out of a total of 209 properties were visited and a full inventory carried out. In the urban areas of San Cristobal and Floreana every property was visited for a full inventory. In the urban areas of Isabela and Santa Cruz, a random sample of five properties was subjected to the full inventory within each town block, while in the remainder of each block, all other properties were visited but only additional alien plant species not recorded for that town block were noted. Results from the urban areas on Santa Cruz (Puerto Ayora) and Isabela (Puerto Villamil) have already been published [17], [18]. We have provided additional details on how properties were sampled in Text S1.

#### Species identification and classification

Plant identification was carried out using the reference collections of the Charles Darwin Research Station herbarium (CDS), floras, published keys, internet resources and expert help. Where possible, identification was carried out to species or subspecies level. Nomenclature follows the Catalogue of the Vascular Plants of Ecuador [19] and the List of known Vascular Plants from the Galapagos Islands [20]. With the property owner's permission, herbarium specimens were collected; otherwise photos were taken and databased.

As each species was recorded, its growth form was allocated to one of the following categories: herb, succulent, vine (climber or creeper), tree (single woody large stem), shrub (multiple woody stems) or subshrub (partly woody stems). In our analyses, the last three categories were grouped as woody above-ground parts. The presence of sexual and/or asexual regeneration, seedlings, flowers and/or fruits was recorded, as well as if the species was under cultivation. Each species was classified as naturalized or not naturalized, to illustrate its behaviour in Galapagos. The naturalized species include both casual alien and naturalized species as defined in [11]. More detailed categorizations follows the Galapagos checklist [20], in which species are recorded as cultivated (introduced for cultivation, not naturalized), accidental (introduced unintentionally, naturalized), doubtfully accidental (introduced, but unknown if intentionally or not, naturalized), doubtfully native (possibly introduced, naturalized) or escaped (originally introduced for cultivation, naturalized). If there was a difference in naturalization state among islands for one species, the classification represents the most advanced state in the naturalization continuum at the level of the whole archipelago. Each species was also assigned one of the following five categories, according to its most common local use: no use, edible, medicinal, ornamental and other (e.g. shade, fence, wood supply). The property was given one of the following land-use categories: commerce, construction, farm, hotel-restaurant, institution, park, private housing and vacant lot.

The data was entered and processed in an Access database. For San Cristobal the largest island where every property was inventoried we present a species accumulation curve [21] using the vegan package [22] performed in R [23].

## Results

### Properties visited

A total of 6031 out of 6233 properties (97%) were visited during the study. In the agricultural humid highlands, a total of 546 (73%) properties were visited and fully surveyed; in the urban dry coastal towns, all 5485 (100%) properties were visited, of which 3416 (62%) were fully surveyed (Table 1).

**Table 1:** Number of properties visited per island and per area, and total surface of each area.

	Rural area		Urban area	
Island	Area (km^2^)	Number of properties visited	Area (km^2^)	Number of properties visited
Floreana	2.9	10 (100%)	0.2	130 (100%)
Isabela	51.6	202 (100%)	0.9	1191 (100%)
San Cristobal	81.0	324 (98%)	3.1	1830 (100%)
Santa Cruz	112.0	10 (10%)	1.4	2334 (100%)
Total	247.4	546 (73%)	5.5	5485 (100%)

### Species composition and characteristics

#### General Checklist composition

A total of 754 alien taxa were recorded, in 468 genera and 123 families. Among these taxa, 723 were identified to species or lower taxonomic levels, 29 were identified as hybrids or cultivars, 20 to genus level and recognized as distinct (Table S1). Two hundred and fifty seven taxa were new records for the Galapagos Islands (Table S1), adding to the previous recording of 511 alien plant taxa for the archipelago. In all, 3023 plant specimens were deposited at the Charles Darwin Research Station herbarium (CDS). The recorded taxa are hereafter referred to as species.

**Figure 2:**
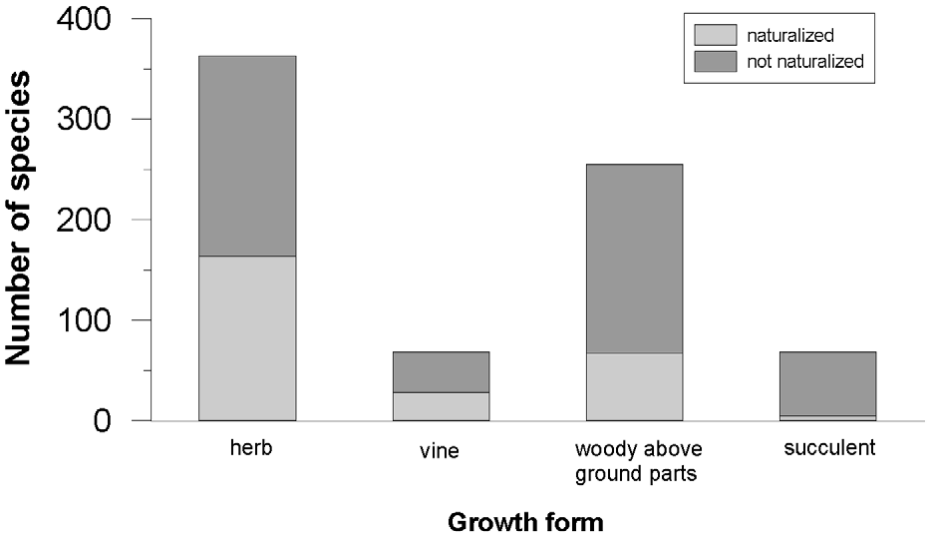
The total number of recorded species per growth form, categorized according to naturalization status.

Dicotyledons represented 73% (554) of all species, monocotyledons 24% (183), and there were 7 gymnosperms and 10 pteridophytes. The five families that contained the highest number of species were Fabaceae (Papilionaceae, Caesalpiniaceae and Mimosaceae) 56 species (including 23 naturalized), Poaceae 50 (36), Asteraceae 42 (26), Cactaceae 31(2), and Solanaceae 29 (16). Poaceae, Asteraceae, Fabaceae and Solanaceae were the families with the highest number of naturalized species, representing about 40% of all species recorded as naturalized. The proportion of naturalized species per family was strongly driven by escaped species in Solanaceae and Fabaceae (81% and 61% respectively) as compared to Poaceae and Asteraceae (33% and 19%). And the proportion of escaped species among intentionally introduced species (including naturalized and non-naturalized species) was 50% in Solanaceae, 46% in Poaceae, 30% in Fabaceae and 24% in Asteraceae. In Cactaceae, only two species naturalized and all the other species were cultivated as ornamentals. Vines and herbaceous growth forms were most likely to establish wild populations. Almost half of the alien species (363) were herbaceous and 45% of these were naturalized (Figure 2).This proportion was not significantly different from that for vines at 41% (*χ* ^2^ = 0.2271, df = 1, p = 0.6637).

In total, 647 species were recorded in the rural areas and 616 species in the urban areas, of which 507 species were shared between both area types. In Floreana, 203 species were recorded, in Isabela 407, in San Cristobal 603 and in Santa Cruz 576. Due to the limited sampling in the Santa Cruz rural area (10 properties out of 209), the real number of alien species is higher on that island; other data from Santa Cruz (including non-inhabited areas) show the total number of alien species to be at least 668, and if survey of the rural area was completed, that number would increase.

#### Most commonly recorded species per area

None of the ten most commonly recorded species in the rural and urban areas were shared between the two areas, and the most common species in the rural area were much more widespread than those in the urban area (Table S1). For example, the most abundant species in the rural area, *Psidium guajava*, was found in over 90% of the properties surveyed, while the most abundant species in the urban area, *Aloe vera*, was only found in 30% of urban properties. It should be noted that in San Cristobal's rural area, *Rubus niveus* ranked first with 293 records (90% of visited properties) whereas it ranked only 21^st^ for the rural areas combined. In the rural area, five out of the ten most common species were edible, one was medicinal and four species had no use; in the urban area, seven of the most common species had a use, i.e. edible (n = 2), medicinal (n = 2) and ornamental (n = 3). In both areas, eight out of the ten species were naturalized.

Seven of the most commonly recorded species (*Bryophyllum pinnatum*, *Oxalis corniculata*, *Passiflora edulis*, *Psidium guajava*, *Sida rhombifolia*, *Stachytarpheta cayennensis and Synedrella nodiflora*) were recorded as not cultivated in the rural area, as compared to four species (*Amaranthus dubius*, *Eleusine indica*, *Portulaca oleracea and Ricinus communis*) in the urban area. Only one species (*Citrus* x *sinensis*) was always recorded as cultivated in the rural area, as compared to three species (*Aloe vera*, *Cocos nucifera* and *Spondias purpurea*) in the urban area.

#### Naturalization status

**Figure 3:**
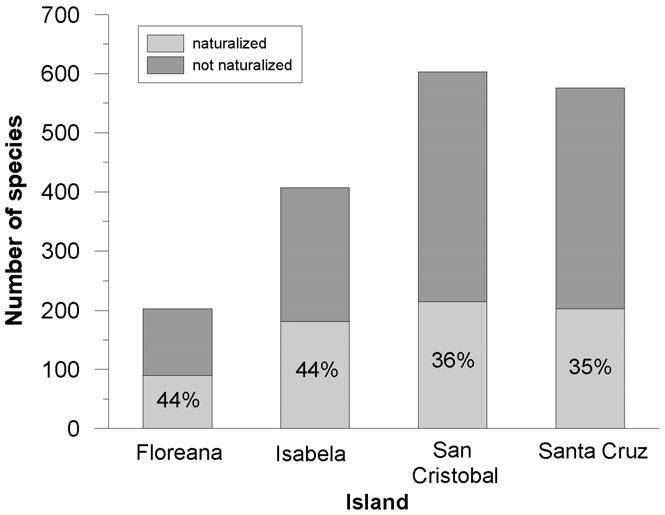
The total number of recorded species per island categorized according to naturalization status.

Out of the 754 species recorded during this study, 264 were recorded as naturalized, of which 52% had escaped from cultivation. The proportion of naturalized species was similar in the rural (37%) and urban area (33%) when data for all islands were combined (*χ* ^2^ = 2.3678, df = 1, p = 0.1239). Floreana and Isabela had the highest proportions of naturalized species per island (44% in both cases) as compared to San Cristobal (36%) and Santa Cruz (35%) (Figure 3).

#### Uses

**Figure 4:**
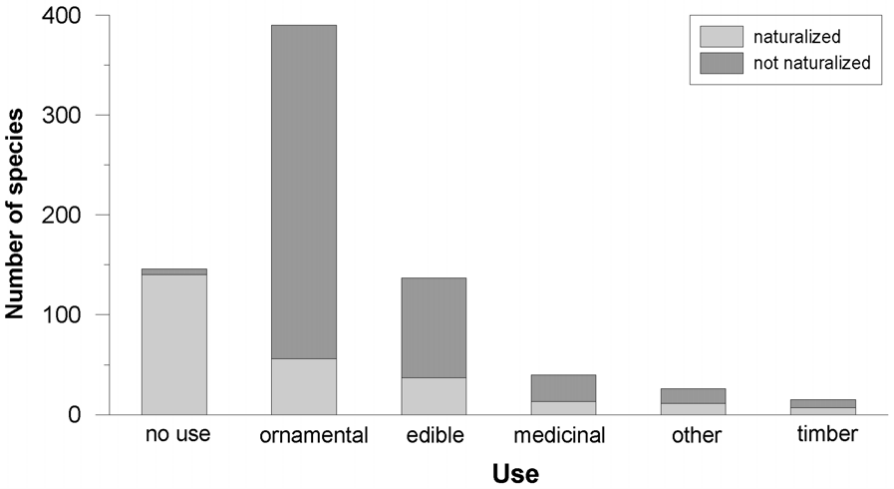
Total number of species per recorded use categorized according to naturalization status.

The majority of species were used as ornamental plants (52%), and a significant proportion was with no use (19%) or used as a food source (18%). Only 14% of the ornamentals were naturalized, compared to 96% for species with no use (Figure 4). On Floreana, only 29% of the species were ornamentals, a much lower proportion than on Isabela (41%), San Cristobal (50%) and Santa Cruz (55%). The proportion of ornamentals in the urban area (58%) was higher than in the rural zone (48%).

### Limitations of the data

The results we present here give a snapshot picture in time of the alien flora of the inhabited areas of the archipelago, whose distribution and composition is continually changing. Properties were only visited once, and depending on the season, annual species may not have not always been apparent, resulting in their under representation in the data set. Also, due to time constraints, the study did not include a complete inventory of every property in the Isabela and Santa Cruz urban areas or in the rural area of Santa Cruz. The latter area had however previously been surveyed, even though incompletely, by Robayo J. and others. Their results have not been included here because they were collected using a different methodology and therefore could not easily be collated with our results. In addition, there are an estimated 25 species that have proved too difficult to identify so far.

## Discussion

### Alien flora outnumbers native flora

This study detected 257 new introduced plant taxa in Galapagos. Prior to this inventory, in 2000, only 511 introduced plant taxa (doubtfully native included) were known from the archipelago, as compared to 552 native taxa (doubtfully native excluded) [24]. Our study reveals a marked dominance of the alien flora over the native flora. Taking into account all of the introduced taxa recorded for Galapagos (870, including records from other studies and doubtfully native species), the alien taxa now outnumber the native ones with a ratio of 1.57∶1 [20]. As described earlier [24], this increase does not reflect an increased recent rate of introduction but rather an increase of the sampling effort, as almost all properties were systematically surveyed. In addition, non-naturalized cultivated species, often previously overlooked, were recorded here.

The alien species reported in this inventory represent 123 families and 468 genera, reflecting a higher diversity than in the Galapagos native flora (97 families, 268 genera). The archipelago's alien and native floras share fifty-five families, of which Fabaceae, Asteraceae and Poaceae are the five most represented in both floras. A similar dominance has been repeatedly reported worldwide for islands [25]–[28], as well as for continents [29]–[32].

Some disharmonies between the Galapagos alien and native floras are to be noted: Cyperaceae, the third-represented family for natives (29 taxa) is only represented by 6 alien taxa. The pteridophytes, an important group in the native flora (105 taxa), are represented by only 9 alien taxa. On the other hand, while there are no native gymnosperms, seven alien taxa were found.

### Most commonly recorded species; a reflection of climate, land use and invasion

The humid rural and arid urban areas do not share any of their ten most commonly recorded species. This reflects the different climatic conditions and the land use history of the two areas. The wet highlands have traditionally been used for agriculture, and half of the commonest alien species in this area are edible species (i.e. *Psidium guajava*, *Passiflora edulis*, *Citrus x sinensis*, *Inga edulis* and *Persea americana*) while none are ornamental. In contrast, the populated coastal towns are located in the arid zones, and one third of the most common species in this region are ornamentals (i.e. *Catharanthus roseus*, *Delonix regia* and *Cocos nucifera*- for which ornamental predominates over edible as use in the urban area).

In rural areas, four of the most commonly recorded species (*Bryophyllum pinnatum*, *Passiflora edulis*, *Psidium guajava and Rubus niveus*), are included in the list of worst invasives for Galapagos [5], as compared to only one species (*Ricinus communis*) for urban areas. All five species were always recorded as not cultivated; the four rural area species were much more widespread than *R. communis* in the urban area. This supports the conclusion of Watson et al. and Snell et al. [33], [34], that the humid highlands are the most degraded climatic zone in Galapagos, due to invasion by alien species, whereas the largely undisturbed, more arid lowlands are not yet experiencing widespread plant invasion.

### Naturalization status

With the addition of the new species detected in this study, 330 species of the Galapagos alien flora are now known to be naturalized, of which 44% have escaped from cultivation. There have been several similar studies on other islands, for example, Easter Island, Desventuradas and Juan Fernández Archipelagos [25], Pacific and Indian Oceans Islands [35], and French overseas islands territories [36], [37]. Even though the sampling effort and the categories considered vary between studies [11], some comparisons with Galapagos can be made. The proportion naturalized here is similar to some other Pacific islands [37], but higher than in Hawaii [38] or New Zealand [39] and lower than in the southeastern Pacific Oceanic Islands [25] (it should be noted that there is a possible bias due to the less complete record of the non-naturalized plants in areas outside of Galapagos).

Another comparison is the ratio of naturalized to native species. In Galapagos, this ratio is 0.59∶1, following [20], a low value compared to many tropical islands [25], [35]. In addition the proportion of escaped species among those intentionally introduced for cultivation is 21%. This may reflect the much more recent colonisation history of Galapagos, whereby many species have not been present long enough to naturalize. This suggests that the number of naturalized species among the current alien flora could increase in the future. This pattern of increase can already be seen within Galapagos. Floreana, the first island to be colonized (and hence the first island to receive plant introductions) and where the number of people has remained fairly stable and low, has the highest proportion of naturalized species but fewer ornamentals than the more recently colonised islands of Santa Cruz and San Cristobal. The human population on the latter two islands has substantially increased in the past 40 years [24], [40], with an increase in the number of houses and thus ornamental gardens. The more recent human colonization date and the higher proportion of ornamentals probably accounts for the lower percentages of naturalization on San Cristobal and Santa Cruz. The relationship among islands and species to number of inhabitants, history and size of area is further discussed by Trueman et al. [41]. In short, propagule pressure has not been particularly high because of the low human population size in Galapagos compared to other islands.

The naturalization proportions for ornamental and edible species were low, whereas almost all of the species with no use (96%) were naturalized. This group was predominantly composed of accidentally introduced herbaceous or doubtfully native species, the latter by definition being integrated into natural systems.

### Survey as a method for early detection of new introductions

Early detection of new introductions and their timely elimination is one of the key steps in the control of invasives [42]. However, while it is logical that species are easier and cheaper to eliminate before they become established, detecting small populations of new introductions is time consuming and difficult [43], with detectability inversely proportional to population size [44]. Policy advisors have thus suggested that effort should be focused on carrying out regular surveys around key sites of introduction such as seaports and airports, as well as areas of high human population or use. For plants, the nursery trade has been shown to be one of the worst culprits for the intentional introduction of new species [45], [46] and advances have been made in several countries to reduce sales of potentially invasive species, both through voluntary methods and legislation [45], [47]. To date, thankfully, there is no nursery trade to speak of in Galapagos. In addition, in many countries there has been increased reliance on voluntary help to detect new introductions. Though in some areas, the quantity of amateur naturalists outnumbers professionals and provides an important source of information [48], in Galapagos, as in other locations, we expect botanists to provide the best likelihood of detecting newly naturalizing species [49]. Awareness raising could help reduce the rate of new species introductions, and perhaps lead the person having introduced a new plant species to alert authorities in case of suspected invasive behavior, as happened in the case of tropical kudzu [50].

The inventory carried out in Galapagos focused on the archipelago's inhabited areas, which are the sources of new introductions, and detected 257 new plant species. Six Ecuadorians were trained in botanical identification in the process. This exhaustive inventory required a total 17 person years (carried out over 5 years by a four person team), and cost an estimated $300,000 USD. This corresponds to an average of $50 USD per property. The species found during this survey were reviewed in terms of their potential for invasiveness, and feasibility of complete eradication from each or all of the four inhabited islands. Invasiveness was assessed on the base of distribution, regeneration patterns in each area, and naturalization and invasiveness in Galapagos and elsewhere. A Galapagos weed risk assessment was developed for all known alien vascular plant species in the archipelago by C. Buddenhagen, A. Tye, P. Pheloung and J. Mader (unpublished data), which assigned an invasion-risk group for each species. This led to identification of key future invasives that were included in eradication feasibility studies carried out by the Charles Darwin Foundation. Among these were for example *Cryptostegia grandiflora* (detected in four gardens), *Acacia nilotica* (nine plants detected over two gardens) and *Aristolochia elegans* (10 plants detected in one property). These three species are known worldwide to be problems and are potentially highly invasive in Galapagos. Further details on these eradication projects and the detected species are presented in [17], [18], [51].

**Figure 5:**
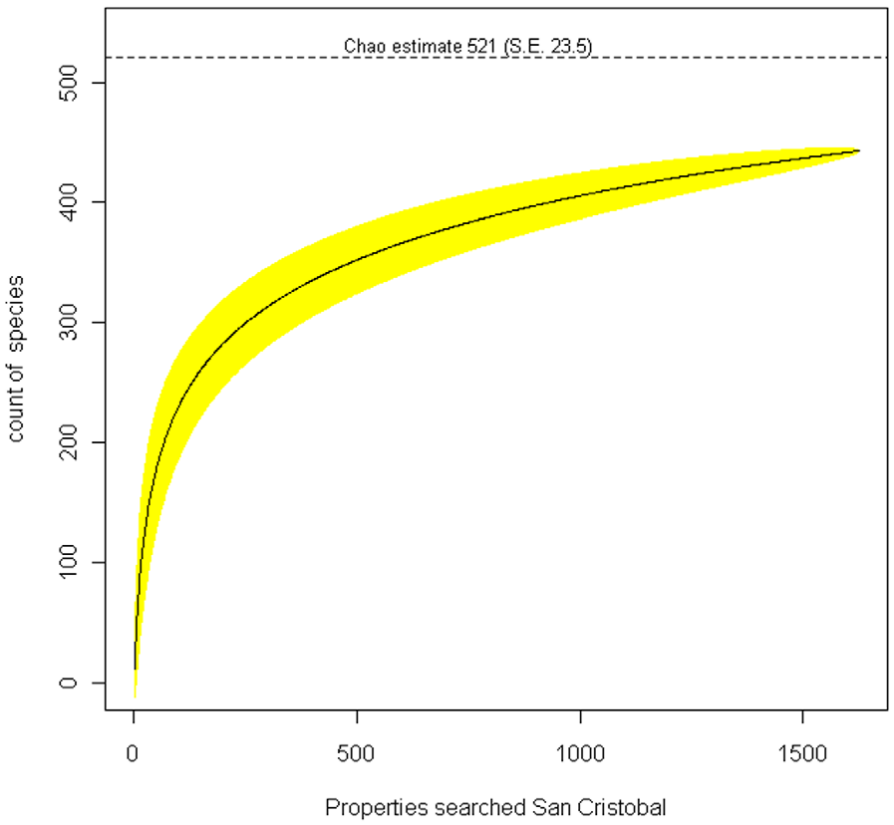
Accumulation curve for alien plant species on San Cristobal, including the Chao estimate of total species richness. The curves include 95% confidence intervals and represent 100 random permutations.

Since 1999, it has been illegal to bring new plant species into the archipelago, yet introductions still happen, stressing the need for stronger quarantine control measures. Once on the island, seedlings and cuttings get moved between gardens via neighbours; few people sell plants and those who do provide only a limited variety of medicinal, edible, and ornamental species (e.g. *Codiaeum variegatum*, *Bougainvillea* sp.). Several native plant nurseries have been set up to provide alternatives to alien ornamentals. Thus, within the inhabited areas, there are no obvious foci of introduction, and a species area curve (Figure 5) clearly shows that in order to detect new introductions, a survey as thorough as the one reported here would need to be repeated if it were to be used as the method for early detection and elimination of new species.

In addition, there is little community action to help identify new introductions in inhabited areas, and as reported in [51], public support for elimination of new species can be difficult to attain. Although there is a fledgling regulation to provide legal support for enforced removal, the precautionary approach is not well appreciated and the regulation has yet to be used.

### Conclusion

This work represents an extensive inventory of the alien flora in Galapagos inhabited areas and is the best dataset of its kind for Galapagos. Besides providing a baseline against which to compare future introductions, it has been used as a basis for weed risk assessments in the archipelago, acts as an early detection tool that allows for potential elimination, and has provided concrete evidence to management agencies of the risks posed by the inhabited areas to future invasion of the Galapagos National Park. In addition, the data have already contributed to worldwide meta analyses that study patterns in the invasion process of island archipelagos e.g. [3], [27]. Finally, the predominance of ornamental plants in the alien flora points to the urgent need for institutional and community awareness and involvement to develop proactive and concerted action for the use of native plants in gardens.

## Supporting Information

Table S1 Complete list of the alien vascular plant taxa encountered in the inhabited areas of Galapagos. Species name: 1 indicates a new record for Galapagos. Introduction status in Galapagos: Ac) Accidental (introduced unintentionally, naturalized); AcQ) Doubtfully accidental (introduced, naturalized but it is not known if introduction was casual or intentional); Cu) Cultivated (introduced for cultivation, not naturalized); Es) Escaped (introduced for cultivation, naturalized); NaQ) Doubtfully native, possibly introduced. Growth form: h) herbaceous; s) succulent; sh) shrub; ssh) subshrub; t) tree; v) vine. Use: edi) edible; med) medicinal; non) no use; orn) ornamental; oth) other; tim) timber. % of visited rural properties and % of urban properties fully surveyed: * indicates one of the ten most common species for each area; nfs): species found only in non-fully surveyed properties; a blank cell indicates that the species was not recorded.
(0.41 MB PDF)

Text S1 Complementary details on data collection.
(0.01 MB PDF)
