# Supplementary material for: An Extensive Alien Plant Inventory from the Inhabited Areas of Galapagos
Source: PLoS One. 2010 Apr 21;5(4):e10276. doi: 10.1371/journal.pone.0010276 (PMC2858082; doi:10.1371/journal.pone.0010276)
Supplement: Table S1 — Complete list of the alien vascular plant taxa encountered in the inhabited areas of Galapagos. Species name: 1 indicates a new record for Galapagos. Introduction status in Galapagos: Ac) Accidental (introduced unintentionally, naturalized); AcQ) Doubtfully accidental (introduced, naturalized but it is not known if introduction was casual or intentional); Cu) Cultivated (introduced for cultivation, not naturalized); Es) Escaped (introduced for cultivation, naturalized); NaQ) Doubtfully native, possibly introduced. Growth form: h) herbaceous; s) succulent; sh) shrub; ssh) subshrub; t) tree; v) vine. Use: edi) edible; med) medicinal; non) no use; orn) ornamental; oth) other; tim) timber. % of visited rural properties and % of urban properties fully surveyed: * indicates one of the ten most common species for each area; nfs): species found only in non-fully surveyed properties; a blank cell indicates that the species was not recorded. (0.41 MB PDF) [file pone.0010276.s001.pdf]

Family	Species name	Common name English (local-Spanish)	Introduction status in Galapagos	Growth form	Use	% of visited rural properties	% of fully surveyed urban properties	Floreana_rural	Isabela_rural	San Cristobal_rural	Santa Cruz_rural	Floreana_urban	Isabela_urban	San Cristobal_urban	Santa Cruz_urban
Acanthaceae	*Asystasia gangetica* (L.) T. Anderson [1]		Cu	h	orn	0.4	0.6			X				X	X
Acanthaceae	*Crossandra infundibuliformes* Nees [1]		Cu	ssh	orn	0.2	0.2			X				X	
Acanthaceae	*Elytraria imbricata* (Vahl) Pers.		Ac	h	non	4.4	0.1		X						X
Acanthaceae	*Fittonia albivenis* (Lindl. ex Veitch) Brummitt [1]		Cu	h	orn		0.1							X	X
Acanthaceae	*Hypoestes phyllostachya* Baker [1]		Cu	h	orn	0.9	0.2			X				X	X
Acanthaceae	*Justicia brandegeana* Wassh. & L.B. Sm. [1]	Shrimp Plant (flor de camarón)	Cu	h	orn	1.3	0.5			X	X			X	
Acanthaceae	*Justicia carnea* Lindl. [1]	(cola de pavo real)	Cu	sh	orn	0.7	0.6			X				X	X
Acanthaceae	*Megaskepasma erythrochlamys* Lindau [1]		Cu	sh	orn		<0.1							X	
Acanthaceae	*Odontonema cuspidatum* (Nees) Kuntze	(lava botellas)	Es	sh	orn	4.2	1.1		X	X	X		X	X	X
Acanthaceae	*Pachystachys lutea* Nees	Golden Shrimp-plant (rabito de gallo, flor de camarón)	Cu	sh	orn	0.7	0.4			X	X			X	X
Acanthaceae	*Pseuderanthemum carruthersii* (Seem.) Guillaumin		Cu	sh	orn	8.4	6.1	X	X	X	X	X	X	X	X
Acanthaceae	*Ruellia malacosperma* Greenm. [1]		Es	h	orn	0.5	0.4		X	X			X		X
Acanthaceae	*Sanchezia speciosa* Leonard [1]		Cu	sh	orn	0.2	<0.1			X				X	
Acanthaceae	*Thunbergia fragrans* Roxb.		Es	v	orn	9.7	0.3			X				X	
Actinidiaceae	*Actinidia deliciosa* (A. Chev.) C.F. Liang & A.R. Ferguson [1]	kiwifruit (kiwi)	Cu	v	edi	0.4				X					
Agavaceae	*Agave americana* L.	Century Plant (cabuya negra, penco)	Cu	s	orn	1.5	0.6		X	X	X		X	X	X
Agavaceae	*Agave angustifolia* var. *marginata* hort. ex Gentry [1]		Cu	s	orn	1.1	1.2		X				X	X	X
Agavaceae	*Agave attenuata* Salm-Dyck [1]		Cu	h	orn	0.2	nfs				X				
Agavaceae	*Furcraea hexapetala* (Jacq.) Urb.	(cabuya, penco blanco)	Es	s	orn	23.4	0.8	X	X	X	X	X	X	X	X
Agavaceae	*Yucca aloifolia* L. [1]		Cu	sh	orn	0.4	0.1			X	X				X
Agavaceae	*Yucca guatemalensis* Baker [1]	(flor de novia, peine de indio)	Cu	sh	orn	15.8	2.5		X	X	X		X	X	X
Aizoaceae	*Aptenia cordifolia* (L. f.) Schwantes [1]		Cu	s	orn	1.5	2.6		X	X		X	X	X	X
Aizoaceae	*Lampranthus aureus* (L.) N.E. Br.		Cu	ssh	orn		0.1								X
Aizoaceae	*Tetragonia tetragonioides* (Pall.) Kuntze [1]	New Zealandspinach (espinaca)	Cu	h	edi	2.9	0.6		X	X			X	X	X
Alliaceae	*Allium cepa* L.	Onion, Spring onion (cebolla paiteña, cebolla blanca)	Cu	h	edi	17.6	1.6		X	X	X	X	X	X	X
Alliaceae	*Allium porrum* L.	Leek (cebolla puerro)	Cu	h	edi	1.1	0.1			X	X				X
Alliaceae	*Allium sativum* L.	Garlic (ajo)	Cu	h	edi	1.3	0.3			X	X	X	X		X

Family	Species name	Common name English (local-Spanish)	Introduction status in Galapagos	Growth form	Use	% of visited rural properties	% of fully surveyed urban properties	Floreana_rural	Isabela_rural	San Cristobal_rural	Santa Cruz_rural	Floreana_urban	Isabela_urban	San Cristobal_urban	Santa Cruz_urban
Alliaceae	*Allium schoenoprasum* L. [1]	Chives (cebollín)	Cu	h	edi	0.7	0.3	X	X		X	X	X	X	X
Amaranthaceae	*Achyranthes aspera* L.		Ac	h	non	36.6	0.1		X	X			X	X	
Amaranthaceae	*Alternanthera mexicana* (Schltdl.) Hieron.	(escansel)	NaQ	h	orn	3.1	0.1		X	X	X			X	X
Amaranthaceae	*Alternanthera sessilis* (L.) R. Br.		Ac	h	non		0.1								X
Amaranthaceae	*Alternanthera tenella* Colla [1]	(pata de paloma)	Cu	h	orn	0.2	0.5		X			X	X		X
Amaranthaceae	*Amaranthus caudatus* L.	(sangorache verde, quinua)	Cu	h	edi	0.4	0.1				X		X	X	
Amaranthaceae	*Amaranthus dubius* Mart. ex Thell.		Ac	h	non	5.7	*22.4	X	X	X		X	X	X	X
Amaranthaceae	*Amaranthus hybridus* L.	(amaranto)	AcQ	h	non	0.4	<0.1		X						X
Amaranthaceae	*Amaranthus lividus* L.		Ac	h	non	6.6	1.8		X	X	X		X	X	X
Amaranthaceae	*Amaranthus spinosus* L.		Ac	h	non	8.2	1.0	X	X	X			X	X	X
Amaranthaceae	*Amaranthus viridis* L.	(amarantus)	AcQ	h	non	1.3	0.5		X	X				X	X
Amaranthaceae	*Celosia argentea argentea* L.	(rabo de conejo)	Es	h	orn	2.0	1.1		X	X	X	X	X	X	X
Amaranthaceae	*Celosia argentea cristata* (L.) Kuntze	(cresta de gallo)	Es	h	orn	0.9	0.5		X	X	X		X	X	X
Amaranthaceae	*Gomphrena globosa* L.		Cu	h	orn		0.1							X	
Amaranthaceae	*Iresine herbstii* Hook. [1]		Cu	h	orn	0.2	0.2				X			X	X
Amaranthaceae	*Iresine lindenii* Van Houtte [1]		Cu	h	orn		<0.1								X
Amaryllidaceae	*Crinum x amabile* var. *augustum* (Roxb.) Ker Gawl.	(amancay, lirio de cinta)	Cu	h	orn	0.7	1.8				X		X	X	X
Amaryllidaceae	*Crinum zeylanicum* (L.) L.		Cu	h	orn	0.7	0.1			X	X			X	X
Amaryllidaceae	*Eucharis* x *grandiflora* Planch. & Linden		Cu	h	orn	3.8	1.9		X	X	X	X	X	X	X
Amaryllidaceae	*Eucrosia bicolor* Ker Gawler		Cu	h	orn	0.4	1.1			X			X	X	
Amaryllidaceae	*Hippeastrum puniceum* (Lam.) Kuntze		Cu	h	orn	5.1	3.0	X	X	X	X	X	X	X	X
Amaryllidaceae	*Hippeastrum reticulatum* Herb. [1]		Cu	h	orn	1.1	0.1			X			X		X
Amaryllidaceae	*Hippeastrum* x Dutch hybrids [1]	Amaryllis (amarilis)	Cu	h	orn	0.2	0.1			X			X		
Amaryllidaceae	*Hymenocallis pedalis* Herb.		Cu	h	orn	4.4	0.8	X		X		X	X	X	X
Amaryllidaceae	*Scadoxus multiflorus* (L.) Friis & Nordal [1]		Cu	h	orn		0.1							X	
Amaryllidaceae	*Zephyranthes rosea* var. *candida* Lindl. [1]		Cu	h	orn	3.5	3.1			X	X		X	X	X
Anacardiaceae	*Mangifera indica* L.	Mango (mango)	Es	t	edi	31.3	13.7	X	X	X	X	X	X	X	X
Anacardiaceae	*Schinus molle* L.	(molle)	Cu	t	orn		0.1					X		X	
Anacardiaceae	*Spondias purpurea* L.	(ciruelo, ovito)	Es	t	edi	56.6	*18.7	X	X	X	X	X	X	X	X
Annonaceae	*Annona cherimola* Mill.	Cherimoya (chirimoya)	Es	sh	edi	12.6	6.0		X	X	X		X	X	X
Annonaceae	*Annona glabra* L.	Pond Apple (anona)	NaQ	t	non		2.2						X		
Annonaceae	*Annona muricata* L.	Soursop (guanábana)	Es	t	edi	11.5	9.4	X	X	X	X	X	X	X	X
Anthericaceae	*Chlorophytum comosum* (Thunb.) Jacques	(mala madre)	Cu	h	orn	2.2	3.0			X	X		X	X	X
Apiaceae	*Anethum graveolens* L.	(eneldo)	Cu	h	med	0.2	<0.1		X					X	
Apiaceae	*Apium graveolens* L.	Celery (apio)	Cu	h	edi	4.9	0.4		X	X	X		X	X	X
Apiaceae	*Coriandrum sativum* L.	Coriander (culantro, cilantro)	Cu	h	edi	14.1	0.9	X	X	X	X	X	X	X	X

Family	Species name	Common name English (local-Spanish)	Introduction status in Galapagos	Growth form	Use	% of visited rural properties	% of fully surveyed urban properties	Floreana_rural	Isabela_rural	San Cristobal_rural	Santa Cruz_rural	Floreana_urban	Isabela_urban	San Cristobal_urban	Santa Cruz_urban
Apiaceae	*Cyclospermum leptophyllum* (Pers.) Sprague ex Britton & P. Wilson	(puccio)	NaQ	h	non	28.9	0.1	X	X	X				X	X
Apiaceae	*Daucus carota* L.	Carrot (zanahoria)	Cu	h	edi	8.6	0.3	X	X	X	X		X	X	X
Apiaceae	*Eryngium foetidum* L. [1]	False Coriander (culantrillo de monte)	Cu	h	edi	2.4	0.2			X				X	X
Apiaceae	*Foeniculum vulgare* Mill.	Fennel (hinojo, lecherillo, eneldo)	Cu	h	med	0.4	<0.1			X	X				X
Apiaceae	*Pastinaca sativa* L.	Parsnip (zanahoria blanca)	Cu	h	edi	1.5	0.7		X	X	X				
Apiaceae	*Petroselinum crispum* (Mill.) A.W. Hill	Parsley (perejil)	Cu	h	edi	5.5	4.1		X	X	X	X	X	X	X
Apocynaceae	*Allamanda cathartica* L.	Yellowbell (campana de oro, copa de oro)	Cu	v	orn	3.3	<0.1		X	X	X		X	X	X
Apocynaceae	*Allamanda schottii* Pohl [1]		Cu	sh	orn		nfs								X
Apocynaceae	*Catharanthus roseus* (L.) G. Don	Madagascar Periwinkle (chavelas)	Es	h	orn	14.8	*17.0	X	X	X	X	X	X	X	X
Apocynaceae	*Mandevilla sanderi* (Hemsl.) Woodson [1]		Cu	ssh	orn		nfs								X
Apocynaceae	*Nerium oleander* L.	Oleander (laurel, laurel de jardín, adelfa)	Cu	sh	orn	5.5	4.9	X	X	X	X	X	X	X	X
Apocynaceae	*Plumeria rubra* L.	Frangipani (flor de María)	Cu	t	orn	2.0	1.7		X	X	X			X	X
Apocynaceae	*Tabernaemontana divaricata* (L.) R. Br. ex Roem. & Schult. [1]		Cu	sh	orn	0.9	0.5			X	X			X	X
Apocynaceae	*Thevetia peruviana* (Pers.) K. Schum.	(jacapa)	Cu	sh	orn	0.5	0.1		X	X	X				X
Araceae	*Acorus gramineus* Aiton [1]		Cu	h	orn	0.2	<0.1			X				X	
Araceae	*Aglaonema commutatum* Schott		Cu	h	orn	1.5	2.7			X	X		X	X	X
Araceae	*Alocasia cucullata* (Lour.) G. Don. [1]		Cu	h	orn		0.1							X	
Araceae	*Alocasia macrorrhizos* (L.) G. Don.	(camacho)	Cu	h	orn	1.8	1.5		X	X	X		X	X	X
Araceae	*Alocasia micholitziana* Sander [1]		Cu	h	orn	0.2	1.0			X				X	
Araceae	*Alocasia plumbea* Van Houtte [1]		Cu	h	orn	2.0	0.1			X	X			X	X
Araceae	*Alocasia sanderiana* hort. ex W. Bull [1]		Cu	h	orn		0.1							X	
Araceae	*Anthurium andraeanum* Linden		Cu	h	orn	2.4	1.2			X	X			X	X
Araceae	*Caladium bicolor* (Aiton) Vent.	Elephant's Ears (corazón de Jesús)	Cu	h	orn	5.5	5.2		X	X	X	X	X	X	X
Araceae	*Caladium humboldtii* Schott [1]		Cu	h	orn		0.6							X	
Araceae	*Colocasia esculenta* (L.) Schott	Taro, Dasheen (papa china, taro)	Es	h	edi	12.6	0.3	X	X	X	X			X	X
Araceae	*Dieffenbachia seguine* (Jacq.) Schott [1]	Dumb Cane (millonaria, chucha)	Cu	h	orn	9.9	9.9		X	X	X	X	X	X	X

Family	Species name	Common name English (local-Spanish)	Introduction status in Galapagos	Growth form	Use	% of visited rural properties	% of fully surveyed urban properties	Floreana_rural	Isabela_rural	San Cristobal_rural	Santa Cruz_rural	Floreana_urban	Isabela_urban	San Cristobal_urban	Santa Cruz_urban
Araceae	*Epipremnum pinnatum* (L.) Engl.	(enredadera, cortina)	Cu	v	orn	3.3	2.4	X	X	X	X	X	X	X	X
Araceae	*Monstera adansonii* var. *laniata* (Schott) Madison [1]		Cu	v	orn		0.1								X
Araceae	*Monstera deliciosa* Liebm.	(costilla de Adán)	Cu	v	orn	0.4	<0.1			X	X			X	X
Araceae	*Monstera obliqua* Miq. [1]		Cu	h	orn	0.2	1.0			X		X		X	X
Araceae	*Philodendron bipinnatifidum* Schott ex Endl. [1]	(costilla de Adán)	Cu	h	orn	0.7	0.8			X	X			X	X
Araceae	*Philodendron erubescens* K. Koch & Augustin [1]		Cu	v	orn	0.5	1.6			X	X			X	X
Araceae	*Philodendron* sp. 1 [1]		Cu	v	orn		nfs								X
Araceae	*Spathiphyllum wallisii* Regel [1]		Cu	h	orn	0.7	0.3			X	X			X	X
Araceae	*Syngonium podophyllum* Schott [1]		Cu	v	orn	3.7	4.4		X	X	X		X	X	X
Araceae	*Syngonium wendlandii* Schott [1]		Cu	h	orn		<0.1							X	
Araceae	*Xanthosoma robustum* Schott.		Cu	h	orn	4.9	0.2			X				X	X
Araceae	*Xanthosoma sagittifolium* (L.) Schott	(otoy)	Es	h	orn	30.4	1.0	X	X	X	X		X	X	X
Araceae	*Zantedeschia aethiopica* (L.) Spreng. [1]	(cartucho)	Cu	h	orn	0.2	0.1		X				X	X	
Araliaceae	*Polyscias cumingiana* (C. Presl.) Fern.-Vill. [1]		Cu	sh	orn	0.2	0.6				X			X	X
Araliaceae	*Polyscias fruticosa* (L.) Harms	(cola de iguana)	Cu	sh	orn	0.2	0.2				X			X	X
Araliaceae	*Polyscias guilfoylei* (W. Bull) L.H. Bailey [1]		Cu	sh	orn	6.8	3.2		X	X	X	X	X	X	X
Araliaceae	*Polyscias scutellaria* Burm. f.) Fosberg [1]		Cu	sh	orn	1.3	1.5		X	X	X		X	X	X
Araliaceae	*Schefflera actinophylla* (Endl.) Harms [1]	(cheflera)	Cu	t	orn	0.2	0.1				X				X
Araliaceae	*Schefflera arboricola* Hayata	(cheflera)	Cu	ssh	orn	0.9	1.7			X	X		X	X	X
Araucariaceae	*Araucaria heterophylla* (Salisb.) Franco [1]		Cu	t	orn	0.4				X	X			X	
Arecaceae	*Bactris gasipaes* Kunth [1]	(palmito)	Cu	t	edi	0.2			X						
Arecaceae	*Caryota mitis* Lour. [1]		Cu	t	orn	0.2					X				
Arecaceae	*Cocos nucifera* L.	(coco, palma)	Cu	t	orn	19.2	*20.4	X	X	X	X	X	X	X	X
Arecaceae	*Dypsis lutescens* (H. Wendl.) Beentje & J. Dransf.	Golden Cane Palm (palma enana)	Cu	t	orn	0.9	2.1		X	X	X		X	X	X
Arecaceae	*Elaeis guineensis* Jacq.	(palma africana)	Cu	t	orn	0.2					X				
Arecaceae	*Phoenix dactylifera* L.	Date Palm (dátil)	Cu	t	orn	0.4	0.2			X	X		X		X
Arecaceae	*Phytelephas aequatorialis* Spruce	(tagua)	Cu	t	orn	1.8				X	X				
Arecaceae	*Pritchardia lanigera* Becc. [1]		Cu	t	orn	0.5	0.2			X	X		X	X	X
Arecaceae	*Ptychosperma elegans* (R. Br.) Blume [1]		Cu	t	orn	1.8	1.0	X	X	X	X		X	X	X
Arecaceae	*Roystonea regia* (Kunth) O.F. Cook [1]	Royal Palm (palma real)	Cu	t	orn	0.9	0.2		X				X	X	X
Arecaceae	*Veitchia merrillii* (Becc.) H.E. Moore [1]		Cu	t	orn		<0.1							X	
Arecaceae	*Washingtonia robusta* H. Wendl. [1]		Cu	t	orn	0.4	0.2				X			X	X
Aristolochiaceae	*Aristolochia odoratissima* L.	(Zaragoza)	Es	v	med	0.4	<0.1				X				X
Asclepiadaceae	*Asclepias curassavica* L.	(mata caballo)	Es	h	orn	21.2	0.1	X	X	X				X	X

Family	Species name	Common name English (local-Spanish)	Introduction status in Galapagos	Growth form	Use	% of visited rural properties	% of fully surveyed urban properties	Floreana_rural	Isabela_rural	San Cristobal_rural	Santa Cruz_rural	Floreana_urban	Isabela_urban	San Cristobal_urban	Santa Cruz_urban
Asclepiadaceae	*Caralluma hesperidum* Maire [1]	(pata de langosta)	Cu	s	orn	0.2	0.1		X				X	X	
Asclepiadaceae	*Cryptostegia grandiflora* (Roxb.) R. Br. [1]	(caucho de la India)	Cu	v	orn		<0.1								X
Asclepiadaceae	*Hoya carnosa* (L. f.) R. Br.	(hoja de cera)	Cu	s	orn	0.5	0.6		X	X	X	X	X	X	X
Asclepiadaceae	*Huernia aspera* N.E. Br. [1]		Cu	s	orn	0.9	1.8		X	X			X	X	X
Asclepiadaceae	*Stapelia gigantea* N.E. Br. [1]		Cu	s	orn		0.1							X	X
Asparagaceae	*Asparagus densiflorus* (Kunth) Jessop [1]	Foxtail fern	Es	v	orn	0.4	0.6				X		X	X	X
Asparagaceae	*Asparagus officinalis* L.	(espárrago)	Cu	v	orn	0.2	<0.1			X					X
Asparagaceae	*Asparagus setaceus* (Kunth) Jessop	Asparagus Fern (crestón)	Es	v	orn	1.5	2.6		X	X	X	X	X	X	X
Asphodelaceae	*Aloe arborescens* Mill. [1]	(sábila de Castilla)	Cu	s	orn	0.4	0.8				X		X	X	X
Asphodelaceae	*Aloe aristata* Haw. [1]		Cu	s	orn	0.5	0.3			X	X			X	X
Asphodelaceae	*Aloe cooperi* Baker [1]		Cu	s	orn		<0.1						X		
Asphodelaceae	*Aloe vera* (L.) Burm. f.	(sábila)	Cu	s	med	19.2	*29.6	X	X	X	X	X	X	X	X
Asphodelaceae	*Haworthia attenuata* (Haw.) Haw. [1]		Cu	s	orn	0.4	0.1			X			X		X
Asteliaceae	*Cordyline fruticosa* (L.) A. Chev.		Cu	sh	orn	4.6	2.3		X	X	X		X	X	X
Asteraceae	*Achillea millefolium* L. [1]	Yarrow	Es	h	orn	0.5	0.1		X	X			X	X	X
Asteraceae	*Acmella ciliata* (HBK) Cassini		AcQ	h	non	0.4				X	X				
Asteraceae	*Acmella sodiroi* (Hieron.) R.K. Jansen		AcQ	h	non	1.5	<0.1			X	X				X
Asteraceae	*Adenostemma platyphyllum* Cass.	(mama Juana, tía Juana)	Es	h	non	19.4	0.2		X	X	X			X	X
Asteraceae	*Ambrosia arborescens* Mill. [1]	(altamisa)	Cu	ssh	med	0.4			X						
Asteraceae	*Ambrosia peruviana* Willd.		Ac	h	med	0.9	0.1			X				X	
Asteraceae	*Bidens cynapiifolia* Kunth		Ac	h	non	0.4				X					
Asteraceae	*Bidens pilosa* L.	(amor seco)	NaQ	h	non	44.0	2.9	X	X	X	X		X	X	X
Asteraceae	*Blumea viscosa* (Mill.) V.M. Badillo [1]		Ac	h	non	0.2	0.5		X				X		X
Asteraceae	*Centratherum punctatum* Cass.		Es	h	orn	28.4	0.6		X	X			X	X	X
Asteraceae	*Chrysanthemum coronarium* L. [1]		Cu	h	orn	0.5	0.1		X	X				X	
Asteraceae	*Conyza bonariensis* (L.) Cronquist		Ac	h	non	9.0	<0.1	X	X	X	X		X		
Asteraceae	*Conyza canadensis* (L.) Cronquist		Ac	h	non	29.7	<0.1		X	X	X		X		
Asteraceae	*Cosmos bipinnatus* Cav.		Cu	h	orn		nfs								X
Asteraceae	*Cyanthillium cinereum* (L.) H. Rob.		AcQ	h	non	10.3	2.7		X	X			X	X	X
Asteraceae	*Cynara cardunculus* L.	Artichoke (alcachofa)	Cu	h	med	0.4				X					
Asteraceae	*Dahlia pinnata* Cav.	Dahlia (dalia)	Cu	h	orn	5.3	1.1		X	X	X	X	X	X	X
Asteraceae	*Dendranthema indicum* (L.) Des Moul. [1]		Cu	h	orn	0.2	<0.1		X					X	
Asteraceae	*Dendranthema* x *grandiflorum* cv peggy Stevens (Ramat.) Kitam.	Chrysanthemum (pomo)	Cu	h	orn	1.8	2.5		X	X			X	X	X
Asteraceae	*Elephantopus mollis* Kunth [1]		Ac	h	non	10.8				X					
Asteraceae	*Emilia sonchifolia* (L.) D.C. [1]		Ac	h	non		<0.1							X	

Family	Species name	Common name English (local-Spanish)	Introduction status in Galapagos	Growth form	Use	% of visited rural properties	% of fully surveyed urban properties	Floreana_rural	Isabela_rural	San Cristobal_rural	Santa Cruz_rural	Floreana_urban	Isabela_urban	San Cristobal_urban	Santa Cruz_urban
Asteraceae	*Erechtites hieraciifolius* var. *cacalioides* (Fisch. ex Spreng.) Griseb.		Ac	h	non	14.8	<0.1		X	X				X	
Asteraceae	*Galinsoga quadriradiata* Ruiz & Pav.	(hierba de cuy)	Ac	h	non	9.0		X	X	X	X				
Asteraceae	*Gamochaeta purpurea* (L.) Cabrera	(puscala)	NaQ	h	non	2.0			X						
Asteraceae	*Gazania rigens* L. (Gaertn.)		Cu	h	orn	0.4	0.3		X	X			X	X	
Asteraceae	*Helianthus annuus* L.	Sunflower (girasol)	Cu	ssh	orn	1.6	1.3		X	X	X		X	X	X
Asteraceae	*Koanophyllon solidaginoides* (Kunth) R.M. King & H. Rob.		NaQ	h	non	3.5			X	X					
Asteraceae	*Lactuca sativa* L.	Lettuce (lechuga)	Cu	h	edi	7.0	0.2	X	X	X	X			X	X
Asteraceae	*Matricaria recutita* L.	Chamomile (manzanilla)	Cu	h	med	2.9	0.1		X	X	X	X	X	X	X
Asteraceae	*Porophyllum ruderale* ssp. *macrocephalum* (DC.) R.R. Johnson	(ruda gallinazo)	Ac	h	non	4.4	5.9		X	X	X	X	X	X	X
Asteraceae	*Pseudelephantopus spicatus* (B. Juss. ex Aubl.) C.F. Baker		Ac	h	non	2.7	0.1	X		X		X			
Asteraceae	*Pseudelephantopus spiralis* (Less.) Cronquist		Ac	h	non	65.2	0.3		X	X	X			X	X
Asteraceae	*Pseudogynoxys scabra* (Benth.) Cuatrec.	(San Juan)	Cu	v	orn	0.2	<0.1			X					X
Asteraceae	*Senecio macroglossus* DC. [1]		Cu	v	orn	0.2	0.2				X			X	X
Asteraceae	*Smallanthus sonchifolius* (Poepp.) H. Rob.	(jicapa)	Cu	ssh	edi	0.7	<0.1		X	X	X			X	
Asteraceae	*Sonchus oleraceus* L.		Ac	h	non	31.3	0.4	X	X	X	X			X	X
Asteraceae	*Synedrella nodiflora* (L.) Gaertn.		Ac	h	non	*77.8	2.2	X	X	X	X	X	X	X	X
Asteraceae	*Tagetes erecta* L.	(flor de muerto, arrayosa, sintzo)	Cu	h	orn	7.9	2.6	X	X	X	X	X	X	X	X
Asteraceae	*Tanacetum parthenium* (L.) Sch. Bip. [1]	Feverfew (Santa María)	Cu	h	med	0.2					X				
Asteraceae	*Taraxacum officinale* Weber	Dandelion (taraxaco, diente de león)	AcQ	h	med	2.2	0.4	X	X	X	X	X	X	X	X
Asteraceae	*Tithonia diversifolia* (Hemsl.) A. Gray	(jícama)	Es	ssh	orn	0.4				X	X				
Asteraceae	*Zinnia peruviana* (L.) L.		Es	h	orn	2.0	1.7			X	X			X	X
Balsaminaceae	*Impatiens balsamina* L.	(mírame lindo)	Es	h	orn	4.8	1.5		X	X	X			X	X
Balsaminaceae	*Impatiens walleriana* Hook. f.	(mírame lindo)	Es	h	orn	6.6	4.4		X	X	X			X	X
Begoniaceae	*Begonia* "aya" [1]	Begonia (begonia)	Cu	h	orn	0.4	0.2			X				X	
Begoniaceae	*Begonia* "haageana"	Begonia (begonia)	Cu	h	orn	0.4				X	X				
Begoniaceae	*Begonia dichroa* Sprague	Begonia (begonia)	Cu	h	orn	3.5	2.4		X	X	X			X	X
Begoniaceae	*Begonia rex* Putz.	King begonia, painted-leaf begonia, begonia rex (begonia)	Cu	h	orn	2.2	0.9			X	X			X	X
Begoniaceae	*Begonia semperflorens* Link & Otto	Bedding begonia, wax	Cu	h	orn	3.3	2.3			X	X	X		X	X

Family	Species name	Common name English (local-Spanish)	Introduction status in Galapagos	Growth form	Use	% of visited rural properties	% of fully surveyed urban properties	Floreana_rural	Isabela_rural	San Cristobal_rural	Santa Cruz_rural	Floreana_urban	Isabela_urban	San Cristobal_urban	Santa Cruz_urban
		begonia (begonia)													
Begoniaceae	*Begonia* x *erythrophylla* Neumann [1]	Begonia (begonia)	Cu	h	orn		0.1								X
Betulaceae	*Betula* cf. *alleghaniensis* Britton [1]		Cu	t	orn	0.2					X				
Bignoniaceae	*Crescentia cujete* L.	Calabash Tree (calabasa, botote pilche, mate, botote)	Cu	sh	oth	6.0	0.1		X	X				X	
Bignoniaceae	*Jacaranda mimosifolia* D. Don [1]	Jacaranda (acacia celeste)	Cu	t	orn	0.7	0.1			X	X			X	X
Bignoniaceae	*Mansoa standleyi* (Steyrm.) A.H. Gentry	(palo de ajo)	Cu	v	edi	0.2					X				
Bignoniaceae	*Spathodea campanulata* P. Beauv.	African Tulip Tree (tulipán africano)	Cu	t	orn	0.2	<0.1				X			X	
Bixaceae	*Bixa orellana* L.	Annatto (achiote)	Es	sh	edi	26.4	0.2	X	X	X	X	X		X	X
Bixaceae	*Cochlospermum vitifolium* (Willd.) Spreng.	(bototillo, capo)	Cu	t	orn	1.3			X						
Bombacaceae	*Ceiba pentandra* (L.) Gaertn.	Cotton Tree (ceibo)	Cu	t	orn	3.5	0.1		X	X	X		X	X	
Bombacaceae	*Matisia cordata* Bonpl.	(zapote)	Cu	t	edi	12.1			X	X	X				
Bombacaceae	*Ochroma pyramidale* (Cav. ex Lam.) Urban	Balsa (balsa)	Es	t	tim	11.4	0.1		X	X	X			X	
Boraginaceae	*Borago officinalis* L. [1]	Borage (borraja, suelda-suelda)	Cu	h	med	0.4			X	X					
Boraginaceae	*Cordia alliodora* (Ruiz & Pav.) Oken	(laurel)	Es	t	oth	5.9		X	X	X	X				
Boraginaceae	*Heliotropium rufipilum* var. *rufipilum* (Benth.) I.M. Johnst.		AcQ	sh	non	0.2					X				
Brassicaceae	*Brassica juncea (L.) Coss.*		Cu	h	edi	0.2				X					
Brassicaceae	*Brassica napus* L.	Oilseed Rape, turnip (nabo)	Es	h	edi	10.3	0.4	X	X	X	X	X	X	X	X
Brassicaceae	*Brassica oleracea* var. *botrytis* L.	Cauliflower (coliflor)	Cu	h	edi	4.0	0.1	X	X	X	X		X		X
Brassicaceae	*Brassica oleracea* var. *capitata* L.	Cabbage (col, col morada)	Cu	h	edi	12.5	0.6	X	X	X	X		X	X	X
Brassicaceae	*Brassica oleracea* var. *gemmifer* (DC.) Zenker	Brussels Sprout (col de Bruselas)	Cu	h	edi	0.2				X					
Brassicaceae	*Brassica oleracea* var. *italica* Plenck	Broccoli (brócoli)	Cu	h	edi	4.9	0.1		X	X	X		X		X
Brassicaceae	*Brassica rapa* L.	(nabo chino)	Es	h	non	20.9	0.5	X	X	X	X		X	X	X
Brassicaceae	*Capsella bursa-pastoris* (L.) Medik.		Ac	h	non	2.4		X	X	X	X				
Brassicaceae	*Cardamine bonariensis* Pers.	(berro)	Es	h	non	3.1	0.1		X	X	X				X
Brassicaceae	*Coronopus didymus* (L.) J.E. Sm.	Lesser Swine-cress (mastuerzo)	Ac	h	non	9.7	0.1	X	X	X	X		X		X
Brassicaceae	*Lepidium sativum* L.		Cu	h	non	0.5			X	X					
Brassicaceae	*Lepidium virginicum* L.		AcQ	h	non	23.1	1.4		X	X				X	X
Brassicaceae	*Matthiola incana* (L.) R. Br. [1]		Cu	h	orn		0.1						X	X	
Brassicaceae	*Nasturtium officinale* R. Br.		Es	h	orn	0.4					X				
Brassicaceae	*Raphanus sativus* L.	Radish (rábano)	Es	h	edi	6.8	0.2	X	X	X	X		X		X

Family	Species name	Common name English (local-Spanish)	Introduction status in Galapagos	Growth form	Use	% of visited rural properties	% of fully surveyed urban properties	Floreana_rural	Isabela_rural	San Cristobal_rural	Santa Cruz_rural	Floreana_urban	Isabela_urban	San Cristobal_urban	Santa Cruz_urban
Bromeliaceae	*Ananas comosus* (L.) Merr.	Pineapple (piña)	Cu	s	edi	28.2	0.9	X	X	X	X	X	X	X	X
Bromeliaceae	*Bromelia pinguin* L.	(piñuela)	Cu	s	oth	4.4			X	X	X				
Bromeliaceae	*Cryptanthus sinuosus* L.B. Sm. [1]		Cu	s	orn		nfs								X
Bromeliaceae	*Neoregelia carolinae* fo. *tricolor* (M.B. Foster) M.B. Foster ex L.B. Sm. [1]		Cu	h	orn	0.4	nfs			X	X				X
Bromeliaceae	*Tillandsia* cf. *pretiosa* Mez [1]	(huaicundo)	Cu	h	orn	0.4				X					
Bromeliaceae	*Tillandsia cyanea* Linden ex K. Koch [1]	(huaicundo)	Cu	h	orn	0.2				X					
Buddlejaceae	*Buddleja davidii* Franch. [1]		Cu	sh	orn	0.2					X				
Cactaceae	*Brasiliopuntia brasiliensis* (Willd.) Haw.		Cu	s	orn	1.3	1.0		X	X	X		X	X	X
Cactaceae	*Cereus peruvianus* var. 1 (L.) J.S. Muell.		Cu	s	orn	2.4	7.5		X	X	X		X	X	X
Cactaceae	*Cereus peruvianus var. monstrosus* DC.		Cu	s	orn	0.7	0.5			X	X		X	X	X
Cactaceae	*Cleistocactus* sp. 1		Cu	s	orn	0.5	0.1		X	X	X				X
Cactaceae	*Disocactus flagelliformis* (L.) Barthlott	(flor de látigo, floricuerno, nopalillo)	Cu	s	orn		0.2							X	X
Cactaceae	*Disocactus* sp. 1	(nopalillo)	Cu	s	orn		nfs								X
Cactaceae	*Echinocactus* sp. 1		Cu	s	orn	0.2					X				
Cactaceae	*Echinopsis calochlora* K. Schum.		Cu	s	orn	0.4	0.5			X	X			X	X
Cactaceae	*Echinopsis eyriesii* (Turpin) Zucc.		Cu	s	orn	0.2	0.2			X			X		X
Cactaceae	*Echinopsis pachanoi* (Britton & Rose) Friedrich & G.D. Rowley		Cu	s	orn		0.1						X		X
Cactaceae	*Epiphyllum oxypetalum* (DC.) Haw.	(galán de noche, reina de la noche)	Cu	s	orn	0.2	1.1		X			X	X	X	X
Cactaceae	*Hatiora gaertneri* (Regel) Barthlott		Cu	s	orn	0.2	0.1				X			X	X
Cactaceae	*Hylocereus polyrhizus* (F.A.C. Weber) Britton & Rose	(flor de cáliz)	Es	ssh	orn	1.3	2.9	X	X	X	X	X	X	X	X
Cactaceae	*Mammillaria backebergiana* F.G. Buchenau		Cu	s	orn		0.1							X	
Cactaceae	*Mammillaria candida* Scheidw.		Cu	s	orn	0.2	0.2				X			X	X
Cactaceae	*Mammillaria columbiana* Salm-Dyck		Cu	s	orn		0.1							X	X
Cactaceae	*Mammillaria prolifera* ssp. 1 (Mill.) Haw.	(el mundo)	Cu	s	orn	0.2	0.2			X				X	X
Cactaceae	*Mammillaria prolifera* ssp. *texana* (Engelm.) Borg.		Cu	s	orn	0.2	<0.1			X					X
Cactaceae	*Mammillaria* sp. 1		Cu	s	orn		<0.1								X
Cactaceae	*Opuntia cf. monacantha* ssp. *monacantha* (Willd.) Haw.		Cu	s	orn	0.9	1.9			X			X	X	X
Cactaceae	*Opuntia cf. monacantha* var. *variegata* (Willd.) Haw.		Cu	s	orn		0.3						X		X
Cactaceae	*Opuntia dillenii* (Ker Gawl.) Haw.		Cu	s	orn	1.3	4.1		X		X		X	X	X
Cactaceae	*Opuntia ficus-indica* (L.) Mill.		Es	s	orn	0.9	2.8			X			X	X	X
Cactaceae	*Opuntia microdasys* var. 1		Cu	s	orn	0.7	0.2		X	X	X		X		X

Family	Species name	Common name English (local-Spanish)	Introduction status in Galapagos	Growth form	Use	% of visited rural properties	% of fully surveyed urban properties	Floreana_rural	Isabela_rural	San Cristobal_rural	Santa Cruz_rural	Floreana_urban	Isabela_urban	San Cristobal_urban	Santa Cruz_urban
	(Lehm.) Pfeiff.														
Cactaceae	*Opuntia microdasys* var. *albispina* Fobe		Cu	s	orn		0.2							X	X
Cactaceae	*Opuntia subulata* (Muehlenpf.) Engelm.		Cu	s	orn		0.1							X	X
Cactaceae	*Pereskia aculeata* Mill. [1]		Cu	s	orn		<0.1								X
Cactaceae	*Pereskia bleo* (Kunth) DC. [1]		Cu	s	orn		<0.1							X	X
Cactaceae	*Schlumbergera truncata* (Haw.) Moran		Cu	s	orn	0.2	nfs				X				X
Cactaceae	*Selenicereus anthonyanus* (Alexander) D.R. Hunt		Cu	s	orn		0.1								X
Cactaceae	*Selenicereus megalanthus* (Schumann ex Vaupel) Moran [1]	yellow pitaya (pitahaya amarilla)	Cu	s	orn	0.2				X					
Caesalpiniaceae	*Bauhinia monandra* Kurz.	Orchid Tree (orquidea del pobre)	Cu	t	orn	3.7	3.2	X	X	X		X	X	X	X
Caesalpiniaceae	*Bauhinia* sp. 1		Cu	t	orn		0.1							X	
Caesalpiniaceae	*Caesalpinia bonduc* (L.) Roxb.	(mora, chililica)	NaQ	sh	non	0.2	0.3				X		X		
Caesalpiniaceae	*Caesalpinia gilliesii* (Wall. ex Hook.) D. Dietr.		Cu	sh	orn		<0.1					X			
Caesalpiniaceae	*Caesalpinia pulcherrima* (L.) Sw.		Es	sh	orn	2.7	2.8		X	X	X	X	X	X	X
Caesalpiniaceae	*Cassia fistula* L.	(chorizo, cigarro)	Cu	t	orn		0.3							X	X
Caesalpiniaceae	*Cassia grandis* L. f.		Es	t	non	1.1				X					
Caesalpiniaceae	*Delonix regia* (Boger ex Hook.) Raf.	Flame Tree, Flamboyant (falsa acacia)	Es	t	orn	12.3	*17.9	X	X	X	X	X	X	X	X
Caesalpiniaceae	*Schizolobium parahyba* (Vell.) S.F. Blake	(pachaco)	Cu	t	orn	0.2					X				
Caesalpiniaceae	*Senna alata* (L.) Roxb.	(palo de abejón)	Es	ssh	orn	1.3	1.5		X	X			X	X	X
Caesalpiniaceae	*Senna bicapsularis* (L.) Roxb.		Ac	ssh	non		0.6								X
Caesalpiniaceae	*Senna obtusifolia* (L.) H.S. Irwin & Barneby	(dormidera)	Ac	h	non	51.1	9.6	X	X	X		X	X	X	X
Caesalpiniaceae	*Senna septemtrionalis* (Viv.) H.S. Irwin & Barneby [1]		Es	sh	non	1.6				X					
Caesalpiniaceae	*Senna siamea* (Lam.) H.S. Irwin & Barneby [1]		Cu	t	orn	4.8	<0.1		X	X				X	X
Caesalpiniaceae	*Tamarindus indica* L.	Tamarind (tamarindo)	Cu	t	edi	2.6	3.3		X	X		X	X	X	X
Campanulaceae	*Hippobroma longiflora* (L.) G. Don		Es	h	non	0.5	<0.1			X					X
Cannabaceae	*Cannabis sativa* L. [1]	Marijuana (marihuana)	Cu	h	oth		nfs								X
Cannaceae	*Canna indica* L.	Indian Shot (achira)	Cu	h	oth	10.4	1.0		X	X	X		X	X	X
Cannaceae	*Canna x generalis* Bailey	(atzera, platanillo)	Es	h	oth	2.9	0.9	X	X	X	X	X	X	X	X
Capparaceae	*Cleome speciosa* Raf. [1]		Es	h	orn	0.2	nfs				X				X
Capparaceae	*Cleome spinosa* Jacq.		Cu	h	orn	0.2	<0.1				X			X	
Capparaceae	*Cleome viscosa* L.		Ac	h	non		1.7							X	X
Caprifoliaceae	*Sambucus nigra* L.	(tilo)	Cu	t	med	2.6	0.1		X	X	X			X	X
Caricaceae	*Carica papaya* L.	Pawpaw, Papaya (papaya)	Es	t	edi	53.8	*25.6	X	X	X	X	X	X	X	X

Family	Species name	Common name English (local-Spanish)	Introduction status in Galapagos	Growth form	Use	% of visited rural properties	% of fully surveyed urban properties	Floreana_rural	Isabela_rural	San Cristobal_rural	Santa Cruz_rural	Floreana_urban	Isabela_urban	San Cristobal_urban	Santa Cruz_urban
Caricaceae	*Carica* x *heilbornii* (Heilborn) V.M. Badillo	(babaco)	Cu	sh	edi	1.1				X					
Caryophyllaceae	*Dianthus caryophyllus* L.	(clavel)	Cu	h	orn	1.5	0.2			X	X			X	X
Caryophyllaceae	*Stellaria media* (L.) Vill.		Ac	h	non	2.4		X	X						
Casuarinaceae	*Casuarina equisetifolia* L.	(árbol de hierro, casuarina)	Cu	t	orn	0.2	<0.1				X				X
Cecropiaceae	*Cecropia* cf. *angustifolia* Trécul [1]		Cu	t	orn	0.4				X	X				
Chenopodiaceae	*Beta vulgaris* var. *cicla* (L.) Koch [1]	Chard (acelga)	Cu	h	edi	4.0	0.1	X	X	X	X		X		X
Chenopodiaceae	*Beta vulgaris* var. *vulgaris* L.	Beetroot (remolacha)	Cu	h	edi	4.6	0.1	X	X	X	X		X	X	X
Chenopodiaceae	*Chenopodium ambrosioides* L.	Wormseed, Mexican Tea (paico)	Es	h	med	10.1	2.1		X	X		X	X	X	X
Chenopodiaceae	*Chenopodium murale* L.		Ac	h	non		0.1					X	X		X
Clusiaceae	*Mammea americana* L. [1]	Mammee Apple (mamey)	Cu	t	edi	0.7	0.1		X	X				X	X
Combretaceae	*Quisqualis indica* L. [1]		Cu	v	orn		nfs								X
Combretaceae	*Terminalia catappa* L.	Indian Almond (almendro)	Cu	t	orn	10.3	7.7	X	X	X	X	X	X	X	X
Commelinaceae	*Callisia fragrans* (Lindl.) Woodson [1]		Cu	h	orn		0.6							X	
Commelinaceae	*Callisia repens* (Jacq.) L. [1]		Cu	h	orn	1.1	1.1			X				X	
Commelinaceae	*Tradescantia fluminensis* Vell. [1]		Es	v	orn	3.5	1.1		X	X	X			X	X
Commelinaceae	*Tradescantia pallida* (Rose) D.R. Hunt		Cu	h	orn	1.3	0.9			X				X	X
Commelinaceae	*Tradescantia* sp. 1 [1]		Cu	h	orn		<0.1								X
Commelinaceae	*Tradescantia* sp. 2 [1]		Cu	h	orn		0.1						X		X
Commelinaceae	*Tradescantia spathacea* Sw.	(barquito de San Pedro, barca de San Pedr)	Cu	h	orn	0.9	2.9			X	X			X	X
Commelinaceae	*Tradescantia zebrina* hort. ex Bosse		Es	h	orn	1.8	1.1			X	X			X	X
Convolvulaceae	*Ipomoea alba* L.		NaQ	v	non	19.8			X						
Convolvulaceae	*Ipomoea batatas* (L.) Lam.	Sweet Potato (camote)	Es	v	edi	26.0	1.6	X	X	X	X	X	X	X	X
Convolvulaceae	*Ipomoea carnea* ssp. *fistulosa* (Mart. ex Choisy) D.F. Austin [1]		Cu	ssh	orn	0.2	0.1			X				X	X
Convolvulaceae	*Ipomoea nil* (L.) Roth		AcQ	v	non	0.5	3.5			X		X		X	X
Convolvulaceae	*Ipomoea quamoclit* L.		Es	v	orn	0.2	0.5		X			X	X	X	X
Convolvulaceae	*Ipomoea tricolor* Cav. [1]		Cu	v	orn	0.4	0.1		X			X		X	X
Convolvulaceae	*Merremia umbellata* (L.) Hallier f.		AcQ	v	non	2.0	0.5			X				X	
Convolvulaceae	*Stictocardia tiliifolia* (Desr.) Hallier f.	(soguilla)	NaQ	v	non	11.2	<0.1		X	X					X
Crassulaceae	*Aeonium* sp. 1 [1]		Cu	s	orn		nfs								X
Crassulaceae	*Bryophyllum daigremontianum* (Raym.-Hamet & H. Perrier) A. Berger [1]	(dulcamara, siempreviva)	Cu	s	orn	1.1	4.8		X	X	X	X	X	X	X
Crassulaceae	*Bryophyllum gastonis-bonnieri* (Raym.-Hamet & H. Perrier) Lauz.-March.	(dulcamara)	Cu	s	orn	2.0	1.7		X	X			X	X	X
Crassulaceae	*Bryophyllum pinnatum* (Lam.) Oken	Mother-of-Thousands (hoja	Es	s	med	*79.3	4.4	X	X	X	X	X	X	X	X

Family	Species name	Common name English (local-Spanish)	Introduction status in Galapagos	Growth form	Use	% of visited rural properties	% of fully surveyed urban properties	Floreana_rural	Isabela_rural	San Cristobal_rural	Santa Cruz_rural	Floreana_urban	Isabela_urban	San Cristobal_urban	Santa Cruz_urban
		del aire)													
Crassulaceae	*Crassula ovata* (Mill.) Druce [1]		Cu	s	orn	0.4	0.5				X			X	X
Crassulaceae	*Echeveria peacockii* Croucher [1]		Cu	s	orn	0.2	<0.1			X			X		
Crassulaceae	*Echeveria pulvinata* Rose [1]		Cu	s	orn		0.1							X	
Crassulaceae	*Graptopetalum paraguayense* (N.E.Br.) E. Walther [1]		Cu	s	orn	0.4	0.4			X				X	
Crassulaceae	*Greenovia aurea* Webb & Berth. [1]		Cu	s	orn	0.2	0.2				X			X	X
Crassulaceae	*Kalanchoe blossfeldiana* Poelln.		Cu	s	orn	6.6	6.1		X	X	X	X	X	X	X
Crassulaceae	*Kalanchoe eriophylla* Hills. & Bojer ex Tul. [1]		Cu	s	orn		<0.1								X
Crassulaceae	*Kalanchoe fedtschenkoi* Ham. Pers.		Cu	s	orn	0.2	0.3		X				X	X	X
Crassulaceae	*Kalanchoe tubiflora* (Harv.) Raym.-Hamet		Es	s	orn	2.4	2.0		X	X	X		X	X	X
Crassulaceae	*Sedum lineare* Thunb. [1]		Cu	s	orn		<0.1								X
Crassulaceae	*Sedum pachyphyllum* Rose [1]		Cu	s	orn		0.1							X	X
Crassulaceae	*Sedum rubrotinctum* R.T. Clausen [1]		Cu	s	orn		<0.1							X	
Cucurbitaceae	*Citrullus lanatus* (Thunb.) Matsun. & Nakai	Watermelon (sandía)	Es	v	edi	10.1	6.8	X	X	X		X	X	X	X
Cucurbitaceae	*Cucumis dipsaceus* Ehrenb. ex Spach	(huevo de tigre)	AcQ	v	non	5.7	7.9	X	X	X			X	X	X
Cucurbitaceae	*Cucumis melo* L.	Melon (melón)	Cu	v	edi	5.7	1.3		X	X		X	X	X	X
Cucurbitaceae	*Cucumis sativus* L.	Cucumber (pepinillo, pepino)	Cu	v	edi	10.8	1.0	X	X	X		X	X	X	X
Cucurbitaceae	*Cucurbita ficifolia* Bouché	Fig-leaf Gourd (zambo)	Cu	v	edi	2.7	0.3		X	X	X			X	X
Cucurbitaceae	*Cucurbita moschata* (Duchesne ex Lam.) Duchesne ex Poir.	Pumpkin, Winter Squash (zapallo)	Cu	v	edi	15.0	1.5		X	X	X		X	X	X
Cucurbitaceae	*Cucurbita pepo* L.	Courgette, Marrow (zucchini)	Cu	v	edi	1.3	0.1	X	X	X			X		
Cucurbitaceae	*Cyclanthera pedata* var. *edulis* (Naudin) Cogn.	(achogcha)	Cu	h	edi	2.9	0.1		X	X	X			X	X
Cucurbitaceae	*Luffa cylindrica* (L.) M. Roem.	Loofa (esponja)	Cu	v	oth		0.3							X	X
Cucurbitaceae	*Momordica charantia* L.	(achogcha China)	Es	v	non	13.4	12.4			X	X			X	X
Cucurbitaceae	*Sicana odorifera* (Vell.) Naudin [1]	(girón, calabacín)	Es	v	edi	0.4			X	X					
Cupressaceae	*Cupressus macrocarpa* Hartw.	(ciprés)	Cu	t	orn	4.0	0.1		X	X	X				X
Cupressaceae	*Juniperus communis* Thunb. [1]	(ciprés)	Cu	t	orn	1.6	<0.1			X					X
Cupressaceae	*Thuja orientalis* L.	(ciprés)	Cu	t	orn	4.0	3.4		X	X	X	X	X	X	X
Cycadaceae	*Cycas circinalis* L. [1]		Cu	t	orn	0.2	0.1				X			X	X
Cycadaceae	*Cycas revoluta* Thunb. [1]		Cu	t	orn	0.5	0.3			X	X			X	X
Cyclanthaceae	*Carludovica palmata* Ruiz & Pav.	Panama Hat Palm (paja toquilla, cade)	Cu	t	orn	4.8	0.3		X	X	X			X	X
Cyperaceae	*Cyperus involucratus* Rottb.		Es	h	orn	1.1	1.8	X		X	X		X	X	X

Family	Species name	Common name English (local-Spanish)	Introduction status in Galapagos	Growth form	Use	% of visited rural properties	% of fully surveyed urban properties	Floreana_rural	Isabela_rural	San Cristobal_rural	Santa Cruz_rural	Floreana_urban	Isabela_urban	San Cristobal_urban	Santa Cruz_urban
Cyperaceae	*Cyperus odoratus* L.		Ac	h	non	28.0	1.3	X	X		X		X	X	X
Cyperaceae	*Cyperus papyrus* L.	(papiro)	Cu	h	orn		<0.1								X
Cyperaceae	*Cyperus rotundus* L.		Ac	h	non	0.4	<0.1			X				X	
Cyperaceae	*Eleocharis geniculata* (L.) Roem. & Schult.		NaQ	h	non		0.3						X		
Cyperaceae	*Rhynchospora radicans* (Schltdl. & Cham.) Pfeiffer		NaQ	h	non	0.2				X					
Davalliaceae	*Nephrolepis cordifolia cordifolia* (L.) C. Presl.		Cu	h	orn	1.1	0.2			X	X		X		X
Davalliaceae	*Nephrolepis exaltata* cv. *Gretnae* (L.) Schott		Cu	h	orn	1.5	3.8		X	X			X	X	X
Davalliaceae	*Nephrolepis exaltata* cv. *Norwoodii* (L.) Schott. [1]		Cu	h	orn	0.7					X		X		
Davalliaceae	*Nephrolepis exaltata* cv. *Smithii* (L.) Schott		Cu	h	orn	0.2	<0.1			X					
Davalliaceae	*Nephrolepis exaltata* cv. *Verona* (L.) Schott [1]		Cu	h	orn	0.2				X					
Davalliaceae	*Nephrolepis pendula* (Raddi) J. Sm.		Cu	h	orn	0.4	<0.1			X	X				X
Dilleniaceae	*Dillenia indica* L.	(leña de índigo)	Cu	t	orn	0.2					X				
Dracaenaceae	*Dracaena angustifolia* Roxb. [1]		Cu	ssh	orn	1.1	1.2		X	X	X		X	X	X
Dracaenaceae	*Dracaena deremensis* Engl. [1]		Cu	ssh	orn	0.9	0.4			X	X		X	X	X
Dracaenaceae	*Dracaena fragrans* (L.) Ker Gawl. [1]		Cu	ssh	orn	0.9	1.2		X	X			X	X	X
Dracaenaceae	*Dracaena marginata* Hort. [1]		Cu	ssh	orn	0.2	0.1				X			X	X
Dracaenaceae	*Dracaena sanderiana* Mast. [1]		Cu	sh	orn		<0.1							X	
Dracaenaceae	*Sansevieria stuckyi* God.-Leb. [1]		Cu	h	orn		0.1							X	
Dracaenaceae	*Sansevieria trifasciata* Prain	Mother-in-law's Tongue (lengua de suegra)	Es	h	orn	6.2	6.5		X	X	X	X	X	X	X
Euphorbiaceae	*Acalypha amentacea wilkesiana* (Müll. Arg.) Fosberg	Beefsteak plant, copperleaf, Jacob's coat (cresta de pavo)	Cu	sh	orn	7.3	1.1		X	X	X		X	X	X
Euphorbiaceae	*Acalypha hispida* Burm. f.	Chenille plant (cola de zorro, rabo de mono)	Cu	sh	orn	1.6	0.3		X	X	X		X	X	X
Euphorbiaceae	*Acalypha marginata* Spreng.		Cu	sh	orn	5.9	0.4		X	X	X		X	X	X
Euphorbiaceae	*Aleurites moluccana* (L.) Willd. [1]	Candlenut (cocochile, árbol de tocte)	Cu	t	oth	1.1	0.1			X				X	
Euphorbiaceae	*Breynia disticha* var. *nivosa* (W. Bull) Croizat	Foliage flower, snowbush (arbolito de Navidad)	Cu	sh	orn	3.7	6.7		X	X			X	X	X
Euphorbiaceae	*Chamaesyce hirta* (L.) Millsp.		Ac	h	non	1.6				X					
Euphorbiaceae	*Chamaesyce ophthalmica* (Pers.) D.G. Burch		Ac	h	non	32.6	14.3	X	X	X	X	X	X	X	X
Euphorbiaceae	*Chamaesyce thymifolia* (L.) Millsp. [1]		Ac	v	non		1.7								X

Family	Species name	Common name English (local-Spanish)	Introduction status in Galapagos	Growth form	Use	% of visited rural properties	% of fully surveyed urban properties	Floreana_rural	Isabela_rural	San Cristobal_rural	Santa Cruz_rural	Floreana_urban	Isabela_urban	San Cristobal_urban	Santa Cruz_urban
Euphorbiaceae	*Cnidoscolus aconitifolius* (Mill.) I.M. Johnst. [1]	Tree Spinach (chaya, saya)	Es	sh	med	3.8	1.7			X	X			X	X
Euphorbiaceae	*Codiaeum variegatum* (L.) A. Juss.	Croton (croton, espelma)	Cu	sh	orn	18.1	12.7	X	X	X	X	X	X	X	X
Euphorbiaceae	*Euphorbia cotinifolia* L.		Cu	sh	orn	0.4	<0.1		X		X				X
Euphorbiaceae	*Euphorbia cyathophora* Murray		Es	h	orn	1.3	1.8		X	X			X	X	X
Euphorbiaceae	*Euphorbia graminea* Jacq. [1]		Ac	h	non	22.0	0.9		X	X			X	X	
Euphorbiaceae	*Euphorbia heterophylla* L.		Es	h	non	0.4	0.6			X			X	X	X
Euphorbiaceae	*Euphorbia lactea* Haw.		Cu	ssh	orn	2.6	3.4	X	X	X	X	X	X	X	X
Euphorbiaceae	*Euphorbia milii* Des Moul.	Crown of Thorns (corona del Señor, corona de Cristo)	Cu	ssh	orn	1.5	1.6		X	X	X		X	X	X
Euphorbiaceae	*Euphorbia pulcherrima* Willd. ex Klotzsch	Poinsettia (flor de Panamá, flor de Pascua)	Cu	sh	orn	2.6	2.0		X	X	X		X	X	X
Euphorbiaceae	*Euphorbia tirucalli* L.		Cu	sh	orn		0.1								X
Euphorbiaceae	*Jatropha curcas* L.	(piñon)	Es	sh	oth	6.8	0.4		X	X		X	X	X	X
Euphorbiaceae	*Jatropha gossypiifolia* L. [1]		Es	sh	non		<0.1								X
Euphorbiaceae	*Jatropha podagrica* Hook. [1]		Cu	ssh	orn	0.4	0.2		X	X				X	X
Euphorbiaceae	*Manihot esculenta* Crantz	Cassava, Manioc (yuca)	Cu	sh	edi	38.3	2.1	X	X	X	X		X	X	X
Euphorbiaceae	*Pedilanthus tithymaloides* (L.) Poit.	(zapatillas rojas)	Cu	ssh	orn	4.9	4.0		X	X	X	X	X	X	X
Euphorbiaceae	*Phyllanthus acidus* (L.) Skeels	(grosella)	Cu	t	edi	3.5	5.3	X	X	X	X	X	X	X	X
Euphorbiaceae	*Ricinus communis* L.	Castor Oil (higuerrilla)	Es	ssh	non	34.2	*20.5	X	X	X	X		X	X	X
Fabaceae	*Abrus precatorius* L.		Es	v	non	0.5	<0.1	X							X
Fabaceae	*Arachis hypogaea* L.	Groundnut, Peanut (maní)	Es	h	edi	1.6	nfs		X	X	X				X
Fabaceae	*Arachis pintoi* Krapov. & W.C. Gregory [1]	Pinto peanut, fodder peanut, perennial peanut (maní forrajero)	Cu	h	non	0.4				X					
Fabaceae	*Cajanus cajan* (L.) Millsp.	Pigeon Pea (fréjol de palo)	Cu	ssh	edi	16.1	2.0	X	X	X	X	X		X	X
Fabaceae	*Canavalia ensiformis* L. [1]	Jack bean (soya)	Cu	v	edi	0.2				X					
Fabaceae	*Centrolobium paraense* Tul.	(amarillo lagarto)	Cu	t	tim	2.7	<0.1		X	X	X			X	
Fabaceae	*Clitoria ternatea* L.		Es	v	orn	0.4	0.3			X				X	X
Fabaceae	*Crotalaria retusa* L.		AcQ	h	non	4.9	1.7			X				X	
Fabaceae	*Desmodium glabrum* (Mill.) DC.		NaQ	h	non		1.5							X	X
Fabaceae	*Desmodium incanum* DC.		NaQ	h	non	65.6	0.1	X	X	X	X			X	
Fabaceae	*Desmodium intortum* (Mill.) Urb.		NaQ	h	non	24.7			X	X	X				
Fabaceae	*Erythrina corallodendron* Herb. Madr. ex Wallich		Cu	t	oth	2.6			X		X				
Fabaceae	*Erythrina edulis* Triana ex Micheli [1]	(guato)	Cu	t	edi	0.2					X				
Fabaceae	*Erythrina fusca* Lour.	(palo prieto, porotillo)	Cu	t	oth	1.1			X						
Fabaceae	*Erythrina poeppigiana* (Walp.) O.F. Cook	(poro gigante)	Cu	t	oth	2.4			X	X	X				

Family	Species name	Common name English (local-Spanish)	Introduction status in Galapagos	Growth form	Use	% of visited rural properties	% of fully surveyed urban properties	Floreana_rural	Isabela_rural	San Cristobal_rural	Santa Cruz_rural	Floreana_urban	Isabela_urban	San Cristobal_urban	Santa Cruz_urban
Fabaceae	*Erythrina smithiana* Krukoff	(porotillo)	Cu	t	oth	30.6	0.1	X	X	X	X		X	X	
Fabaceae	*Geoffroea spinosa* Jacq.	(seca)	NaQ	t	orn	0.4	0.6	X				X			
Fabaceae	*Gliricidia sepium* (Jacq.) Kunth ex Walp.	(mata ratón, madero negro, nacedero)	Cu	t	oth	6.0	0.4	X	X	X	X	X	X	X	X
Fabaceae	*Lablab purpureus* (L.) Sweet	(zarandajo)	Es	v	edi	4.6	2.7	X	X	X	X	X		X	X
Fabaceae	*Macroptilium lathyroides* (L.) Urb.		Es	h	non	0.9	11.7	X		X		X		X	
Fabaceae	*Medicago sativa* L.	Alfalfa, Lucerne (alfalfa)	Cu	h	med	2.9	<0.1		X	X	X		X		
Fabaceae	*Phaseolus coccineus* L. [1]		Cu	v	edi	0.2				X					
Fabaceae	*Phaseolus lunatus* L. [1]	Butter Bean, Lima Bean (habichuela, fréjol de mata)	Cu	v	edi	4.6	0.3	X	X	X		X	X	X	X
Fabaceae	*Phaseolus vulgaris* L.	(fréjol, vainita)	Cu	v	edi	19.0	0.9	X	X	X	X		X	X	X
Fabaceae	*Pisum sativum* L.	Pea (arveja, alverja)	Cu	v	edi	5.5	<0.1	X	X	X	X				X
Fabaceae	*Spartium junceum* L. [1]	(retama)	Cu	sh	orn	0.2			X						
Fabaceae	*Trifolium* cf. *repens* L.	Clover (trébol)	Cu	h	oth	0.2					X				
Fabaceae	*Vicia faba* L.	Broad Bean (haba)	Cu	h	edi	2.4	0.3	X	X	X		X		X	X
Fabaceae	*Vigna unguiculata* (L.) Walp. [1]	Cowpea, Black-eyed Bean (verdura, fréjol chileno)	Cu	v	edi	3.7	0.8		X	X	X		X	X	X
Fabaceae	*Zornia curvata* Mohlenbr.		Ac	h	non	1.6				X					
Flacourtiaceae	*Muntingia calabura* L.	(niguito)	Es	t	orn	0.5	0.1			X	X			X	X
Flacourtiaceae	*Xylosma flexuosa* (Kunth) Hemsl.	(cereza)	Es	t	edi	1.6			X	X	X				
Geraniaceae	*Pelargonium graveolens* L'Hér. [1]	(malva olorosa, esencia de rosa)	Cu	h	med	0.5	0.2		X		X		X		X
Geraniaceae	*Pelargonium peltatum* (L.) L'Hér. [1]	(geranio)	Cu	h	orn		<0.1								X
Geraniaceae	*Pelargonium* x *domesticum* L.H. Bailey [1]	Geranium (malva olorosa)	Cu	h	orn		<0.1								X
Geraniaceae	*Pelargonium* x *hortorum* L.H. Bailey	Geranium (geranio)	Cu	h	orn	7.3	9.3	X	X	X	X	X	X	X	X
Gesneriaceae	*Episcia cupreata* (Hook.) Hanst.	(lazo de amor)	Cu	h	orn	0.9	1.1			X	X			X	X
Gesneriaceae	*Gloxinia perennis* (L.) Fritsch [1]		Cu	h	orn	0.4	0.1			X				X	
Gesneriaceae	*Gloxinia sylvatica* (Kunth) Wiehler [1]		Cu	h	orn	0.4	0.1		X					X	
Gesneriaceae	*Kohleria amabilis* (Planch. & Lindl.) Fritsch		Cu	h	orn	1.5	nfs			X					X
Gesneriaceae	*Kohleria bogotensis* (G. Nicholson) Fritsch		Cu	h	orn	1.1	1.5			X				X	X
Gesneriaceae	*Saintpaulia* cultivars	African Violet (violeta africana)	Cu	h	orn	0.4	0.3			X				X	X
Gesneriaceae	*Sinningia speciosa* (Lodd.) Hiern [1]		Cu	h	orn		0.1							X	X
Heliconiaceae	*Heliconia latispatha* Benth. [1]		Cu	h	orn	0.5	0.1			X				X	
Heliconiaceae	*Heliconia* sp. 1 [1]		Cu	h	orn		nfs								X
Hydrangeaceae	*Hydrangea macrophylla* (Thunb.) DC.	Hydrangea (Hortensia)	Cu	ssh	orn	4.2	0.6		X	X	X		X	X	X
Hydrocharitaceae	*Hydrocharis morsus-ranae* L. [1]		Cu	h	orn		nfs								X

Family	Species name	Common name English (local-Spanish)	Introduction status in Galapagos	Growth form	Use	% of visited rural properties	% of fully surveyed urban properties	Floreana_rural	Isabela_rural	San Cristobal_rural	Santa Cruz_rural	Floreana_urban	Isabela_urban	San Cristobal_urban	Santa Cruz_urban
Iridaceae	*Crocosmia* x *crocosmiiflora* (Lemoine) N.E. Br. [1]		Cu	h	orn	1.1	nfs			X	X				X
Iridaceae	*Eleutherine bulbosa* (Mill.) Urb. [1]	(vara de justicia)	Cu	h	orn	3.5	4.8		X	X	X	X	X	X	X
Iridaceae	*Gladiolus communis* L.	Gladiolus (gladiolo)	Cu	h	orn	1.1	0.3			X	X			X	X
Iridaceae	*Neomarica gracilis* (Herb.) Sprague [1]	(mano poderosa)	Cu	h	orn	1.8	0.6			X	X		X	X	X
Iridaceae	*Sisyrinchium micranthum* Cav.		NaQ	h	non	2.6			X	X					
Juglandaceae	*Juglans neotropica* Diels	(nogal)	Es	t	tim	28.8		X	X	X	X				
Lamiaceae	*Glechoma hederacea* L. [1]	(alivia dolor, cura todo)	Cu	h	med	0.4	<0.1		X				X		
Lamiaceae	*Hyptis mutabilis* (Rich.) Briq.	(tres filos)	AcQ	h	non	32.1	<0.1		X						X
Lamiaceae	*Hyptis pectinata* (L.) Doit.	(poleo)	Ac	h	non	2.6	1.8	X	X	X	X				X
Lamiaceae	*Hyptis rhomboidea* M. Martens & Galeotti		Ac	h	non	2.9			X		X				
Lamiaceae	*Hyptis sidifolia* (L'Hér.) Briq.		Ac	h	non	17.0	0.1			X	X				X
Lamiaceae	*Hyptis* sp. 1		Es	h	non	0.4				X					
Lamiaceae	*Melissa officinalis* L.	Lemon Balm (toronjil)	Cu	h	med	2.7	0.2			X	X			X	X
Lamiaceae	*Mentha suaveolens* Ehrh. [1]		Cu	h	med		nfs								X
Lamiaceae	*Mentha* x *piperita* L.	Peppermint (menta, hierba buena)	Es	h	med	10.3	4.1		X	X	X	X	X	X	X
Lamiaceae	*Ocimum basilicum* var. *basilicum* L.	Basil (albaca)	Cu	h	edi	10.4	7.4		X	X		X	X	X	X
Lamiaceae	*Ocimum basilicum* var. *pilosum* (Willd.) Benth. [1]		Cu	h	edi	0.7	<0.1				X				X
Lamiaceae	*Ocimum campechianum* Mill.	(albahaca morada)	Cu	h	edi	4.2	0.1		X	X	X			X	X
Lamiaceae	*Origanum vulgare* L.	Orégano (orégano)	Cu	h	edi	3.5	0.6		X	X	X		X	X	X
Lamiaceae	*Plectranthus coleoides* Benth.	(barba de viejo)	Cu	h	orn	0.4				X	X				
Lamiaceae	*Plectranthus unguentarius* Codd [1]	(oreganón, orégano grande)	Es	h	edi	11.4	7.1		X	X	X	X	X	X	X
Lamiaceae	*Plectranthus verticillatus* (L. f.) Druce [1]	(planta moneda)	Cu	h	orn	0.4	0.6			X				X	X
Lamiaceae	*Rosmarinus officinalis* L.	Rosemary (romero)	Cu	h	med	0.4				X	X				
Lamiaceae	*Salvia leucantha* L.	(terciopelo, boca de león)	Cu	h	orn	0.2	0.1		X						X
Lamiaceae	*Salvia sagittata* Ruiz & Pav.	(matico, salvia real)	Es	h	med	0.4				X	X				
Lamiaceae	*Scutellaria* sp. 1		Cu	h	med	0.5				X					
Lamiaceae	*Solenostemon scutellarioides* (L.) Codd	(coleos)	Cu	h	orn	6.0	2.7		X	X	X		X	X	X
Lamiaceae	*Stachys agraria* Cham. & Schltdl.		Ac	h	orn	0.2					X				
Lamiaceae	*Stachys arvensis* L.		Ac	h	non	1.5			X						
Lauraceae	*Persea americana* Mill.	Avocado (aguacate)	Es	t	edi	*69.8	0.8	X	X	X	X	X		X	X
Liliaceae	*Hemerocallis* hybrids [1]	(espiga de san Antonio)	Cu	h	orn	0.5	0.3			X	X		X	X	X
Liliaceae	*Ledebouria socialis* (Bak.) Jessop [1]		Cu	h	orn		<0.1								X

Family	Species name	Common name English (local-Spanish)	Introduction status in Galapagos	Growth form	Use	% of visited rural properties	% of fully surveyed urban properties	Floreana_rural	Isabela_rural	San Cristobal_rural	Santa Cruz_rural	Floreana_urban	Isabela_urban	San Cristobal_urban	Santa Cruz_urban
Liliaceae	*Lilium candidum* L. [1]	Madonna Lily (nardo, azucena)	Cu	h	orn	0.4	0.1				X			X	X
Linaceae	*Linum usitatissimum* L. [1]	Flax (linaza)	Cu	h	orn		nfs								X
Lythraceae	*Cuphea bustamanta* Lex. [1]		Cu	ssh	orn	0.2				X					
Lythraceae	*Cuphea hyssopifolia* Kunth [1]		Cu	h	orn	0.2	0.1				X			X	
Lythraceae	*Cuphea racemosa* (L. f.) Spreng.		Ac	h	non	0.4				X	X				
Lythraceae	*Lagerstroemia indica* L.		Cu	t	orn	2.6	0.5			X				X	
Lythraceae	*Lawsonia inermis* L. [1]	Henna (reseda)	Cu	sh	orn		0.3						X		
Malpighiaceae	*Banisteriopsis caapi* (Spruce ex Griseb.) C.V. Morton [1]	(ayahuasca)	Cu	v	med	0.4				X	X				
Malpighiaceae	*Bunchosia cornifolia* Kunth [1]	Barbados Cherry (Nicaragua, Canadá, cererza serrana)	Cu	sh	orn	1.6	0.5			X	X		X	X	X
Malpighiaceae	*Bunchosia* sp. 1		Cu	t	edi	0.7				X	X				
Malpighiaceae	*Bunchosia* sp. 2		Cu	t	edi	0.7				X					
Malpighiaceae	*Galphimia gracilis* Bartl. [1]		Cu	sh	orn		nfs								X
Malpighiaceae	*Malpighia coccigera* L. [1]		Cu	sh	edi	0.2	<0.1				X			X	
Malpighiaceae	*Malpighia emarginata* Sessé & Moc. ex DC. [1]		Cu	t	edi	0.7	<0.1			X	X		X		X
Malvaceae	*Abutilon dianthum* C. Presl. [1]		Es	h	non		<0.1						X		
Malvaceae	*Anoda acerifolia* Cav.		AcQ	h	non	2.0	<0.1	X			X				X
Malvaceae	*Gossypium barbadense* L.	Sea Island Cotton (algodón)	Cu	sh	orn	0.9	0.3			X				X	X
Malvaceae	*Hibiscus mutabilis* L. [1]		Cu	sh	orn		<0.1								X
Malvaceae	*Hibiscus radiatus* Cav. [1]	Monarch Rosemallow (amapola)	Es	h	orn	0.5	0.6		X	X			X	X	X
Malvaceae	*Hibiscus rosa-sinensis* var. *schizopetalus* Dyer	(peregrino)	Cu	sh	orn	31.0	13.5			X	X		X		X
Malvaceae	*Hibiscus rosa-sinensis* L.	(peregrina)	Es	sh	orn	1.3	0.1	X	X	X	X	X	X	X	X
Malvaceae	*Malachra alceifolia* Jacq.		Ac	h	non		3.2							X	
Malvaceae	*Malachra capitata* (L.) L.		Ac	h	non	0.2				X					
Malvaceae	*Malva parviflora* L.	(malva)	AcQ	h	med	0.5	nfs	X		X	X				X
Malvaceae	*Malvastrum americanum* (L.) Torr.		Ac	h	non		0.1								X
Malvaceae	*Malvastrum coromandelianum* (L.) Garcke	(escoba de bruja)	Ac	h	non	26.0	8.6	X	X	X		X	X	X	X
Malvaceae	*Malvastrum tomentosum tomentosum* (L.) S.R. Hill		Ac	h	non		0.1								X
Malvaceae	*Malvaviscus arboreus* Cav.		Cu	sh	orn	14.7	1.1	X	X	X	X	X	X	X	X
Malvaceae	*Sida acuta* Burm. f.	(escoba negra)	Ac	h	non	4.8	0.2	X	X	X	X	X	X		X
Malvaceae	*Sida ciliaris* L.		Ac	h	non		16.7					X	X	X	X
Malvaceae	*Sida rhombifolia* L.	(escobilla)	Ac	h	non	*72.9	1.0	X	X	X	X	X	X	X	X
Malvaceae	*Sidastrum paniculatum* (L.) Fryxell		Ac	ssh	non	7.5	6.2	X		X		X		X	

Family	Species name	Common name English (local-Spanish)	Introduction status in Galapagos	Growth form	Use	% of visited rural properties	% of fully surveyed urban properties	Floreana_rural	Isabela_rural	San Cristobal_rural	Santa Cruz_rural	Floreana_urban	Isabela_urban	San Cristobal_urban	Santa Cruz_urban
Marantaceae	*Calathea allouia* (Aubl.) Lindl. [1]	Guinea Arrowroot (tapioca)	Cu	h	edi	0.2	0.6				X			X	X
Marantaceae	*Calathea majestica* (Linden) H. Kenn. [1]		Cu	h	orn	0.4	0.1			X	X			X	
Marantaceae	*Calathea picturata* K. Koch & Linden [1]		Cu	h	orn		0.1							X	X
Marantaceae	*Calathea zebrina* (Sims) Lindel [1]	(planta cebra)	Cu	h	orn	0.5	0.8			X	X			X	X
Marantaceae	*Ctenanthe setosa* Eichler		Cu	h	orn	2.2	0.9			X	X			X	X
Marantaceae	*Maranta arundinacea* L. [1]		Cu	h	orn	0.2	0.4			X				X	
Meliaceae	*Cedrela odorata* L.	Cuban Cedar, Cigar-box Cedar (cedrela)	Es	t	tim	65.9	0.6	X	X	X	X			X	X
Meliaceae	*Melia azedarach* L.	Chinaberry, Persian Lilac (jasmín de Arabia, san Jacinto)	Es	t	orn	7.7	1.4	X	X	X		X	X	X	X
Meliaceae	*Swietenia macrophylla* King	Mahogany (caoba)	Cu	t	tim	5.1	0.2		X	X	X		X	X	X
Meliaceae	*Swietenia mahagoni* Jacq. [1]		Cu	t	orn		nfs								X
Mimosaceae	*Acacia caven* (Molina) Molina [1]		Cu	t	non	0.2	0.5			X				X	
Mimosaceae	*Acacia nilotica* (L.) Willd. ex Delile	Gum arabic tree, thorn mimosa, Egyptian thorn (acacia espinosa)	Es	t	orn		<0.1								X
Mimosaceae	*Calliandra calothyrsus* Meissner (C Am.)		Cu	t	orn	0.2		X							
Mimosaceae	*Calliandra* cf. *tergemina* (L.) Benth. [1]		Cu	sh	orn	0.2	<0.1				X			X	
Mimosaceae	*Inga edulis* Mart.	(guava bejuco, guava de mico)	Cu	t	edi	*70.3	2.0	X	X	X	X	X	X	X	X
Mimosaceae	*Inga spectabilis* (Vahl) Willd.	(guava machete)	Es	t	edi	46.7	1.0	X	X	X		X		X	
Mimosaceae	*Inga striata* Benth.	(guaba de Castilla, guaba de caja)	Cu	t	edi	2.9	0.1		X	X	X		X		X
Mimosaceae	*Inga vera* Willd. [1]	(guava)	Cu	t	edi	0.4				X					
Mimosaceae	*Leucaena leucocephala* ssp. *glabrata* (Rose) Zárate	(leucaena, ipel ipel)	Es	sh	non	5.9	6.1	X	X	X	X	X	X	X	X
Mimosaceae	*Leucaena trichodes* (Jacq.) Benth.		Es	t	orn	0.2	0.6		X						X
Mimosaceae	*Pseudosamanea guachapele* (Kunth) Harms	(guachapelí)	Cu	t	orn	10.4	0.1			X				X	X
Moraceae	*Artocarpus altilis* (Parkinson) Fosberg	Breadfruit (frutipan, árbol de pan)	Cu	t	edi	3.5	<0.1		X	X	X			X	
Moraceae	*Artocarpus heterophyllus* Lam. [1]		Cu	t	edi		<0.1							X	
Moraceae	*Ficus benjamina* L. [1]	(ficus)	Cu	t	orn	8.2	5.4		X	X	X		X	X	X
Moraceae	*Ficus carica* L.	Edible Fig (higo)	Cu	sh	edi	5.7	1.2	X	X	X		X	X	X	X
Moraceae	*Ficus cf. benghalensis* L.		Cu	t	orn	0.4			X						

Family	Species name	Common name English (local-Spanish)	Introduction status in Galapagos	Growth form	Use	% of visited rural properties	% of fully surveyed urban properties	Floreana_rural	Isabela_rural	San Cristobal_rural	Santa Cruz_rural	Floreana_urban	Isabela_urban	San Cristobal_urban	Santa Cruz_urban
Moraceae	*Ficus elastica* Roxb. ex Hornem.	(caucho)	Cu	t	orn	1.8	0.2		X	X	X		X	X	X
Moraceae	*Ficus pumila* L. [1]		Es	v	orn		<0.1								X
Musaceae	*Musa acuminata* "Guineo enano" Colla	(guineo enano)	Cu	h	edi	51.3	5.7	X	X	X				X	
Musaceae	*Musa acuminata* "Guineo morado" AAA Colla	(guineo morado, guineo rojo)	Cu	h	edi	13.7	0.3	X	X	X				X	
Musaceae	*Musa acuminata* "Guineo" Colla	Banana (guineo)	Cu	h	edi	6.4	0.1	X	X	X	X		X	X	X
Musaceae	*Musa acuminata* "Orito" Colla	Banana (orito)	Cu	h	edi	11.2	<0.1	X	X	X	X			X	X
Musaceae	*Musa acuminata* x *balbisiana* "Maqueño" AAB Colla [1]	(maqueño)	Cu	h	edi	26.4	0.7	X	X	X					
Musaceae	*Musa acuminata* x *balbisiana* "Verde" Colla	Plantain (verde, barraganete, dominico, repe)	Cu	h	edi	4.6		X	X	X		X		X	X
Musaceae	*Musa velutina* H. Wendl & Drude [1]	(guineo de jardín)	Cu	h	orn	0.4			X						
Musaceae	*Ravenala madagascariensis* Sonn. [1]		Cu	t	orn		<0.1								X
Myrtaceae	*Callistemon citrinus* (Curtis) Skeels	Bottlebrush (cepillo)	Cu	t	orn		0.1							X	X
Myrtaceae	*Eucalyptus globulus* Labill.	(eucalipto)	Cu	t	med	0.5	<0.1		X	X				X	
Myrtaceae	*Eugenia stipitata* Mc Vaugh [1]	(cinco de junio, azará, membrillo.)	Cu	sh	orn		<0.1							X	
Myrtaceae	*Psidium guajava* L.	Guava (guayabo, guayaba)	Es	t	edi	*90.7	3.9	X	X	X	X	X	X	X	X
Myrtaceae	*Syzygium jambos* (L.) Alston	Rose-apple (pomarosa)	Es	t	edi	42.5		X	X	X	X				
Myrtaceae	*Syzygium malaccense* (L.) Merr. & L.M. Perry	(pera noruega)	Es	t	edi	23.6	0.2	X	X	X	X			X	X
Nyctaginaceae	*Bougainvillea* hybrid cultivars	(buganvilla, veranera)	Cu	v	orn		0.5								X
Nyctaginaceae	*Bougainvillea spectabilis* var. *glabra* (Choisy) Hook.	Bougainvillea (buganvilla, veranera)	Cu	v	orn	1.5	0.4		X	X				X	X
Nyctaginaceae	*Bougainvillea spectabilis* var. *spectabilis* Willd.	Bougainvillea (buganvilla, veranera)	Cu	v	orn		0.1								X
Nyctaginaceae	*Bougainvillea* x *buttiana* Holttum & Standl.	(buganvilla, veranera)	Cu	sh	orn	4.6	4.0	X	X	X	X	X	X	X	X
Nyctaginaceae	*Mirabilis jalapa* L.	Marvel of Peru, Four O'Clock Plant (buenas tardes)	Es	h	orn	16.1	3.5		X	X	X		X	X	X
Nymphaeaceae	*Nymphaea capensis* var. *zanzibarensis* (Casp.) Conard [1]		Cu	h	orn		nfs								X
Oleaceae	*Chionanthus pubescens* Kunth	(arupo)	Cu	t	orn	0.4				X	X				
Oleaceae	*Fraxinus uhdei* (Wenz.) Lingelsh. [1]		Cu	t	non	0.2				X					
Oleaceae	*Jasminum azoricum* L. [1]		Cu	v	orn		nfs								X
Oleaceae	*Jasminum grandiflorum* L.		Cu	v	orn	0.5	0.6			X	X			X	
Oleaceae	*Jasminum sambac* (L.) Aiton [1]		Cu	sh	orn		<0.1							X	

Family	Species name	Common name English (local-Spanish)	Introduction status in Galapagos	Growth form	Use	% of visited rural properties	% of fully surveyed urban properties	Floreana_rural	Isabela_rural	San Cristobal_rural	Santa Cruz_rural	Floreana_urban	Isabela_urban	San Cristobal_urban	Santa Cruz_urban
Oleaceae	*Olea europaea* L. [1]	Olive (oliva)	Cu	t	edi	0.2			X						
Orchidaceae	*Cattleya maxima* Lindl.		Cu	h	orn	0.4	nfs				X				X
Orchidaceae	*Stanhopea jenischiana* Kramer ex Rchb. f.	(torito, cabeza de toro)	Cu	h	orn	0.5					X				
Oxalidaceae	*Averrhoa carambola* L. [1]	starfruit (carambola)	Cu	t	edi	0.9				X					
Oxalidaceae	*Oxalis corniculata* L.		AcQ	h	non	*73.4	4.5	X	X	X	X	X	X	X	X
Oxalidaceae	*Oxalis corymbosa* DC.		Es	h	non	46.0	4.9	X	X	X	X	X		X	X
Oxalidaceae	*Oxalis latifolia* Kunth [1]		AcQ	h	orn	0.7	<0.1				X				X
Oxalidaceae	*Oxalis triangularis* A. St.-Hil. [1]		Cu	h	orn		0.1							X	
Oxalidaceae	*Oxalis tuberosa* Molina [1]	(oca)	Cu	h	edi		<0.1								X
Pandanaceae	*Pandanus tectorius* var. *sinensis* Warb. in Engl. [1]		Cu	sh	orn	0.7	0.7			X	X			X	X
Papaveraceae	*Argemone mexicana* L.		Es	h	non	7.0	1.4			X				X	
Passifloraceae	*Passiflora edulis* Sims	Maracuya (maracuyá)	Es	v	edi	*82.1	6.6	X	X	X	X	X	X	X	X
Passifloraceae	*Passiflora ligularis* Juss.	(granadilla)	Es	v	edi	13.7			X	X	X				
Passifloraceae	*Passiflora maliformis* L. [1]	(chalupa)	Es	v	edi	1.3				X					
Passifloraceae	*Passiflora quadrangularis* L.	(badea)	Es	v	edi	12.8	1.1	X	X	X				X	X
Phytolaccaceae	*Phytolacca americana* L. [1]	(ginseng)	Cu	ssh	med	1.1	<0.1			X				X	
Pinaceae	*Pinus radiata* D. Don	(pino)	Cu	t	orn	0.5				X	X				
Piperaceae	*Peperomia fraseri* C. DC. [1]		Cu	h	orn	0.2	0.1			X				X	
Piperaceae	*Peperomia macrostachya* (Vahl) A. Dietr. [1]		Cu	s	orn	1.1	0.4			X	X				X
Piperaceae	*Peperomia obtusifolia* (L.) A. Dietr. [1]		Cu	s	orn	0.4	1.5			X		X		X	X
Piperaceae	*Peperomia sp. 2* [1]		Cu	h	orn		<0.1						X		
Piperaceae	*Piper aduncum* L.		Es	sh	orn	1.3	<0.1		X	X	X			X	
Piperaceae	*Piper peltatum* L.	(cachimuela, Santa María)	Es	h	non	17.6			X		X				
Plantaginaceae	*Plantago major* L.	Greater Plantain (llantén)	Ac	h	med	33.9	8.7	X	X	X	X	X	X	X	X
Plumbaginaceae	*Plumbago auriculata* Lam. [1]		Cu	h	orn	0.2	0.2			X				X	X
Poaceae	*Arthrostylidium pubescens* Rupr. [1]		Cu	sh	non	0.2					X				
Poaceae	*Arundo donax* L. [1]	(carrizo)	Cu	h	orn	2.6		X	X	X	X				
Poaceae	*Axonopus compressus* (Sw.) P. Beauv.		Ac	h	non	15.0				X					
Poaceae	*Axonopus micay* García-Barr.	(micay)	Ac	h	oth	0.7			X	X					
Poaceae	*Bambusa sp. 1* [1]	(caña)	Cu	t	tim	0.7				X					
Poaceae	*Bambusa vulgaris* var. *vittata* Rivière & C. Rivière [1]	Yellow bamboo (caña amarilla)	Cu	t	orn	2.7	<0.1			X				X	
Poaceae	*Bambusa vulgaris* var. *vulgaris* [1]	bamboo (caña verde)	Cu	t	tim	2.2				X					
Poaceae	*Cenchrus brownii* Roem. & Schult. [1]		Ac	h	non	0.2	nfs			X					X
Poaceae	*Cenchrus echinatus* L.		Ac	h	non	1.8	6.6		X	X			X	X	
Poaceae	*Chloris inflata* Link		AcQ	h	non	1.5	15.4	X		X				X	X
Poaceae	*Chloris radiata* (L.) Sw.		NaQ	h	non	1.5	0.8		X	X	X				X
Poaceae	*Coix lacryma-jobi* L.	Job's Tears (lagrima de san Pedro)	Es	h	non	1.3				X					

Family	Species name	Common name English (local-Spanish)	Introduction status in Galapagos	Growth form	Use	% of visited rural properties	% of fully surveyed urban properties	Floreana_rural	Isabela_rural	San Cristobal_rural	Santa Cruz_rural	Floreana_urban	Isabela_urban	San Cristobal_urban	Santa Cruz_urban
Poaceae	*Cymbopogon citratus* (DC.) Stapf	Lemon Grass (hierba Luisa)	Cu	h	med	28.4	11.7	X	X	X	X	X	X	X	X
Poaceae	*Cynodon dactylon* (L.) Pers.		Es	h	oth	6.0	2.2	X	X	X	X	X	X	X	X
Poaceae	*Cynodon nlemfuensis* Vanderyst	(pasto estrella)	Ac	h	oth	9.0				X					
Poaceae	*Dactyloctenium aegyptium* (L.) Willd.		Ac	h	non		0.5						X	X	X
Poaceae	*Digitaria ciliaris* (Retz.) Koeler		NaQ	h	non	2.7	1.6	X		X		X	X	X	X
Poaceae	*Digitaria eriantha* Steud.	(pasto pangola)	Es	h	oth	1.3	0.3			X	X				X
Poaceae	*Digitaria horizontalis* Willd.		Ac	h	non	65.8	2.5	X	X	X	X	X	X	X	X
Poaceae	*Digitaria setigera* Roth		Ac	h	non	39.0	0.5	X	X	X	X		X	X	X
Poaceae	*Echinochloa colona* (L.) Link		Ac	h	non		0.1							X	
Poaceae	*Echinochloa crus-galli* (L.) P. Beauv.		Es	h	non	0.2	0.1			X					X
Poaceae	*Eleusine indica* (L.) Gaertn.		Ac	h	non	61.4	*20.1	X	X	X	X	X	X	X	X
Poaceae	*Eragrostis amabilis* (L.) Wight & Arn. ex Nees		Ac	h	non	3.5	4.7		X	X			X	X	X
Poaceae	*Eragrostis cilianensis* (All.) Vignolo ex Janch.		NaQ	h	non	0.2				X					
Poaceae	*Eragrostis tenuifolia* (A. Rich.) Hochst. ex Steud.		Ac	h	non	3.1	2.3		X					X	
Poaceae	*Guadua angustifolia* Kunth	Bamboo (guadua, caña brava)	Es	t	tim	20.7	0.1	X	X	X	X			X	
Poaceae	*Gynerium sagittatum* (Aublet) P. Beauv.	Saw Grass (caña brava)	AcQ	h	oth	0.2				X					
Poaceae	*Ichnanthus nemorosus* (Sw.) Döll		NaQ	h	non	3.3			X						
Poaceae	*Leptochloa mucronata* (Michx.) Kunth		NaQ	h	non		<0.1								X
Poaceae	*Lolium perenne* L. [1]		Es	h	non	0.4				X					
Poaceae	*Melinis minutiflora* P. Beauv.	Molasses Grass (pasto miel)	Es	h	non	4.9				X	X				
Poaceae	*Oplismenus setarius* (Lam.) Roem. & Schult		NaQ	h	non	35.2	<0.1		X	X	X				X
Poaceae	*Oryza sativa* L.	Rice (arróz)	Cu	h	edi		<0.1								X
Poaceae	*Panicum maximum* Jacq.	Guinea Grass (pasto sabolla, pasto Tanzania)	Es	h	oth	24.7	1.6	X	X	X	X			X	X
Poaceae	*Panicum polygonatum* Schrad.		Ac	h	non	0.5				X	X				
Poaceae	*Paspalum conjugatum* Bergius	Sourgrass (pasto de burro)	NaQ	h	non	65.0	0.6	X	X	X	X			X	X
Poaceae	*Pennisetum purpureum* Schumach.	Elephant Grass (pasto elefante)	Es	h	oth	62.1	0.2	X	X	X	X		X	X	X
Poaceae	*Phyllostachys aurea* Rivière & C. Rivière [1]		Cu	h	orn	0.4					X				
Poaceae	*Poa annua* L.		AcQ	h	non	0.2			X						
Poaceae	*Pogonatherum paniceum* (Lam.) Hack. [1]	(bambú enano)	Cu	h	orn	0.4	0.4			X				X	X
Poaceae	*Saccharum officinarum* L.	Sugarcane (caña de azúcar)	Cu	h	edi	25.6	1.3	X	X	X	X	X	X	X	X

Family	Species name	Common name English (local-Spanish)	Introduction status in Galapagos	Growth form	Use	% of visited rural properties	% of fully surveyed urban properties	Floreana_rural	Isabela_rural	San Cristobal_rural	Santa Cruz_rural	Floreana_urban	Isabela_urban	San Cristobal_urban	Santa Cruz_urban
Poaceae	*Setaria sphacelata* (Scumach.) Moss	(pasto miel (en San Cristóbal))	Cu	h	oth	7.5				X	X				
Poaceae	*Sorghum arundinaceum* (Desv.) Stapf	(sorgo)	Cu	h	non	0.2	0.5			X				X	X
Poaceae	*Sporobolus tenuissimus* (Schrank) Kuntze		Ac	h	non	11.5				X					
Poaceae	*Urochloa brizantha* x *ruziziensis* [1]	(pasto Mulato)	Cu	h	oth	0.5				X					
Poaceae	*Urochloa decumbens* (Stapf) R.D. Webster	(braquiaria)	Es	h	oth	7.0			X	X	X				
Poaceae	*Urochloa mutica* (Forssk.) T.Q. Nguyen	Pará Grass (janeiro)	Es	h	non	0.7	0.1			X	X				X
Poaceae	*Zea mays* L.	Maize (maiz)	Cu	h	edi	23.6	1.5	X	X	X	X		X	X	X
Poaceae	*Zoysia matrella* var. *pacifica* Goudwaard	(césped chino)	Es	h	orn	1.6	8.4		X	X			X	X	X
Polemoniaceae	*Phlox drummondii* Hook.		Es	h	orn	0.2	0.1				X			X	
Polygonaceae	*Antigonon leptopus* Hook. & Arn.	Coral Vine (corazón bello)	Es	v	orn	1.3	0.8	X	X	X	X	X	X	X	X
Polygonaceae	*Triplaris cumingiana* Fisch. & C.A. Mey. ex C.A. Mey.	(Fernansánchez)	Cu	t	tim	9.0			X	X					
Polypodiaceae	*Platycerium* sp. 1 [1]		Cu	h	orn	0.2	0.2				X			X	X
Portulacaceae	*Portulaca grandiflora* Hook.	(beso de un día, flor de un día)	Cu	s	orn	2.9	5.2		X	X	X	X	X	X	X
Portulacaceae	*Portulaca oleracea* L.	Purslane (verdolaga)	NaQ	s	med	19.6	*27.2	X	X	X	X		X	X	X
Portulacaceae	*Portulaca pilosa* L. [1]		Cu	s	orn	0.9	0.4		X	X		X	X	X	X
Portulacaceae	*Portulaca umbraticola* Kunth	(amor del día)	Cu	s	orn	4.8	2.9		X	X	X	X	X	X	X
Portulacaceae	*Portulacaria afra* L. (Jacq.) [1]		Cu	ssh	orn	0.2					X				
Portulacaceae	*Talinum paniculatum* (Jacq.) Gaertn.		Ac	h	non	15.4	0.7			X				X	X
Proteaceae	*Grevillea robusta* A. Cunn. ex R. Br.		Cu	t	oth	1.3	<0.1			X				X	
Pteridaceae	*Adiantum tenerum* Sw. [1]		Cu	h	orn		nfs								X
Pteridaceae	*Pteris ensiformis* Burm. f. [1]		Cu	h	orn	0.2				X					
Punicaceae	*Punica granatum* L.	Pomegranate (granada)	Cu	sh	edi	3.1	1.5		X	X	X	X	X	X	X
Ranunculaceae	*Anemone coronaria* L.		Cu	h	orn	0.2					X				
Rosaceae	*Eriobotrya japonica* (Thunb.) Lindl.	Loquat (níspero)	Es	t	edi	44.1	<0.1	X	X	X	X			X	
Rosaceae	*Fragaria vesca* L.	Strawberry (fresa)	Cu	h	edi	1.3	0.1			X				X	
Rosaceae	*Malus pumila* (L.) Mill.	Apple (manzana)	Cu	t	edi	4.0			X	X					
Rosaceae	*Prunus domestica* L. [1]	Plum (reina Claudia)	Cu	t	edi	0.5			X						
Rosaceae	*Prunus persica* (L.) Batsch	Peach (durazno)	Cu	sh	edi	0.4				X					
Rosaceae	*Prunus serotina* ssp. *capuli* (Cav.) McVaugh	(capulí)	Cu	t	edi	0.2				X					
Rosaceae	*Prunus* sp. 1 [1]		Cu	t	edi	0.4			X						
Rosaceae	*Prunus* sp. 2 [1]	(cereza)	Cu	t	edi	0.4			X		X				
Rosaceae	*Pyrus communis* L.	Pear (pera)	Cu	t	edi	0.4				X	X				
Rosaceae	*Rosa* hybrid cultivars	Rose (rosa)	Cu	ssh	orn	18.9	6.7	X	X	X	X	X	X	X	X
Rosaceae	*Rubus glaucus* Benth.	(mora de Castilla)	Es	sh	edi		<0.1							X	
Rosaceae	*Rubus niveus* Thunb.	(mora)	Es	sh	edi	54.8	0.1		X	X	X			X	X

Family	Species name	Common name English (local-Spanish)	Introduction status in Galapagos	Growth form	Use	% of visited rural properties	% of fully surveyed urban properties	Floreana_rural	Isabela_rural	San Cristobal_rural	Santa Cruz_rural	Floreana_urban	Isabela_urban	San Cristobal_urban	Santa Cruz_urban
Rosaceae	*Rubus ulmifolius* Schott.		Es	sh	non	0.2			X						
Rubiaceae	*Cinchona pubescens* Vahl	Red Quinine (cascarilla, cinchona)	Es	t	tim	0.9				X	X				
Rubiaceae	*Coffea arabica* L.	Coffee (café)	Es	sh	edi	62.5	<0.1	X	X	X	X			X	X
Rubiaceae	*Gardenia augusta* (L.) Merr. [1]	Gardenia (gardenia)	Cu	sh	orn	0.4	<0.1				X				X
Rubiaceae	*Ixora casei* Hance [1]		Cu	sh	orn		nfs								X
Rubiaceae	*Ixora coccinea* L. [1]		Cu	sh	orn	0.7	0.9			X	X			X	X
Rubiaceae	*Ixora finlaysoniana* Wall. ex G. Don [1]		Cu	sh	orn		0.1								X
Rubiaceae	*Morinda citrifolia* L. [1]	(noni)	Cu	ssh	med	1.6	0.5			X				X	
Rubiaceae	*Mussaenda philippica* A. Rich. [1]		Cu	sh	orn		0.3							X	
Rubiaceae	*Pentas lanceolata* (Forssk.) Deflers [1]		Cu	h	orn		<0.1							X	
Rubiaceae	*Posoqueria coriacea* ssp. *formosa* (H. Karst.) Steyerm. [1]		Cu	t	orn	0.2					X				
Rutaceae	*Citrus medica* L.	(citrón)	Cu	t	edi	26.9	0.4		X	X	X	X	X	X	X
Rutaceae	*Citrus reticulata* Blanco	Tangerine, Mandarin (mandarina fina)	Cu	t	edi	39.7	0.8	X	X	X	X	X	X	X	X
Rutaceae	*Citrus* x "limon-mandarina" [1]	(limón-mandarina)	Cu	t	edi	6.0	0.2		X	X			X		
Rutaceae	*Citrus* x "naranja-lima" [1]		Cu	t	edi	0.2			X						
Rutaceae	*Citrus* x *aurantiifolia* (Christm.) Swingle	Lime (limón, limón verde)	Es	t	edi	3.8	0.4		X	X			X	X	X
Rutaceae	*Citrus* x *aurantium* L.	Seville Orange (naranja amarga)	Cu	t	edi	3.7	0.3	X	X	X	X				X
Rutaceae	*Citrus* x *limetta* Risso	Sweet Lime (lima, limón dulce)	Es	t	edi	16.1	0.1	X	X	X	X			X	
Rutaceae	*Citrus* x *limon* (L.) Osbeck	Lemon (limón amarillo, limón sútil)	Es	t	edi	18.5	1.3	X	X	X	X	X	X	X	X
Rutaceae	*Citrus* x *nobilis* Lour.	(mandarina, tangor)	Cu	t	edi	12.6	<0.1			X					X
Rutaceae	*Citrus* x *paradisi* Macfad.	Grapefruit (toronja)	Cu	t	edi	18.9	0.2	X	X	X	X		X	X	X
Rutaceae	*Citrus* x *sinensis* (L.) Osbeck	Sweet Orange (naranja dulce)	Cu	t	edi	*76.6	2.0	X	X	X	X	X	X	X	X
Rutaceae	*Murraya paniculata* (L.) Jack [1]	(flor de ramo)	Cu	sh	orn	0.4	nfs				X				X
Rutaceae	*Ruta graveolens* L.	Rue (ruda)	Cu	ssh	med	14.8	5.1		X	X	X	X	X	X	X
Sapindaceae	*Melicoccus bijugatus* Jacq. [1]	(mamón)	Es	t	orn	0.7			X						
Sapindaceae	*Sapindus saponaria* L.	Soapberry (jaboncillo)	Es	t	tim	21.6		X	X	X					
Sapotaceae	*Chrysophyllum argenteum ssp. panamense* (Pittier) T.D. Penn.	(caimito)	Cu	t	edi	9.9			X	X					
Sapotaceae	*Chrysophyllum cainito* L.	(caimito, mamey)	Cu	t	edi	0.2					X				
Sapotaceae	*Pouteria sapota* (Jacq.) H.E. Moore & Stearn [1]	(mamey serrano)	Cu	t	edi	0.5				X					
Scrophulariaceae	*Angelonia gardneri* Hook. [1]		Cu	h	orn		nfs								X
Scrophulariaceae	*Antirrhinum majus* L.	Snapdragon (perritos)	Cu	h	orn		<0.1							X	

Family	Species name	Common name English (local-Spanish)	Introduction status in Galapagos	Growth form	Use	% of visited rural properties	% of fully surveyed urban properties	Floreana_rural	Isabela_rural	San Cristobal_rural	Santa Cruz_rural	Floreana_urban	Isabela_urban	San Cristobal_urban	Santa Cruz_urban
Scrophulariaceae	*Lindernia* sp. 1	(té verde)	Cu	h	orn		0.4							X	
Scrophulariaceae	*Russelia equisetiformis* Schltdl. & Cham.	(lluvia de fuego, lluvia de coral)	Cu	sh	orn	3.5	1.1		X	X	X		X	X	X
Scrophulariaceae	*Stemodia verticillata* (Mill.) Hassl.		AcQ	h	non	1.8				X					
Scrophulariaceae	*Veronica persica* Poir.		Ac	h	non	4.6		X	X	X	X				
Selaginellaceae	*Selaginella oaxacana* Spring [1]		Cu	h	orn	0.4				X					
Solanaceae	*Browallia americana* L.		AcQ	h	non	8.4	0.2	X	X	X	X			X	X
Solanaceae	*Brugmansia suaveolens* (Humb. & Bonpl. ex Willd.) Bercht. & J. Presl [1]	(floripondio)	Cu	sh	orn	0.2	<0.1			X					X
Solanaceae	*Brugmansia versicolor* Lagerh. [1]	(floripondio, campana)	Es	sh	orn	14.1	1.1	X	X	X	X			X	X
Solanaceae	*Brugmansia* x *candida* Pers.	(guanto, floripondio)	Es	sh	orn	19.2	0.3			X			X		X
Solanaceae	*Brunfelsia grandiflora* D. Don	(variable)	Cu	sh	orn	5.7	0.1		X	X	X			X	X
Solanaceae	*Capsicum annuum* L.	Sweet Pepper (pimiento)	Cu	h	edi	16.1	3.7		X	X	X	X	X	X	X
Solanaceae	*Capsicum baccatum* var. *pendulum* Willd. [1]		Es	h	edi	0.5	nfs		X						X
Solanaceae	*Capsicum frutescens* L.	Chili (ají)	Es	h	edi	23.4	4.5	X	X	X	X		X	X	X
Solanaceae	*Cestrum auriculatum* L'Hér.	(sauco, dama olorosa, puta)	Es	sh	non	1.1					X				
Solanaceae	*Cestrum nocturnum* L.	Queen of the Night (galán de noche)	Cu	sh	orn	0.4	0.2		X					X	
Solanaceae	*Datura inoxia* Mill.		Es	ssh	orn		<0.1						X		
Solanaceae	*Datura metel* L. [1]		Cu	h	orn		0.2						X		X
Solanaceae	*Datura stramonium* var. *stramonium* L.	(chamico)	Es	ssh	non	0.4			X						
Solanaceae	*Datura stramonium* var. *tatula* (L.) Torr.		Es	ssh	orn	0.2	0.1				X				X
Solanaceae	*Nicandra physalodes* (L.) Gaertn.		Ac	ssh	non	13.4	0.2	X	X	X				X	
Solanaceae	*Nicotiana tabacum* L.	Tobacco (tabaco)	Es	h	non	8.4	0.1	X	X	X	X	X		X	X
Solanaceae	*Petunia hybrida* Vilm.	(petunia)	Cu	h	orn		<0.1							X	X
Solanaceae	*Physalis peruviana* L.	(uvilla)	Es	h	edi	0.5			X	X					
Solanaceae	*Solandra grandiflora* Sw.		Cu	v	orn	1.1	0.7		X	X	X			X	X
Solanaceae	*Solanum americanum* Mill.	Black Nightshade (hierba mora)	NaQ	h	med	52.4	7.8	X	X	X	X	X	X	X	X
Solanaceae	*Solanum betaceum* Cav. [1]	Tree Tomato, Tomatillo (tomate de árbol)	Cu	sh	edi	18.9	0.2	X	X	X	X		X	X	X
Solanaceae	*Solanum capsicoides* All. [1]	(tomatillo de montaña, falsa naranjilla)	Cu	h	orn	1.5	0.1		X	X			X		
Solanaceae	*Solanum lycopersicum* L.	Tomato (tomate riñón)	Es	h	edi	26.2	9.0	X	X	X	X	X	X	X	X
Solanaceae	*Solanum melongena* L.	Aubergine, Garden Egg, Eggplant (berenjena)	Cu	sh	edi	2.4	0.1		X	X	X		X		X

Family	Species name	Common name English (local-Spanish)	Introduction status in Galapagos	Growth form	Use	% of visited rural properties	% of fully surveyed urban properties	Floreana_rural	Isabela_rural	San Cristobal_rural	Santa Cruz_rural	Floreana_urban	Isabela_urban	San Cristobal_urban	Santa Cruz_urban
Solanaceae	*Solanum muricatum* Aiton [1]	(pepino dulce)	Cu	ssh	edi	0.4				X					
Solanaceae	*Solanum pimpinellifolium* L.		Es	h	non	7.0	5.5		X	X	X		X	X	X
Solanaceae	*Solanum quitoense* Lam.	Lulo (naranjilla)	Es	sh	edi	29.1	1.3	X	X	X	X		X	X	X
Solanaceae	*Solanum tuberosum* L.	Potato (papa)	Cu	h	edi	11.4	0.5	X	X	X	X		X	X	X
Solanaceae	*Solanum wendlandii* Hook. f. [1]		Cu	v	orn		0.1							X	
Sterculiaceae	*Guazuma ulmifolia* Lam.	(guasmo)	Cu	t	tim	0.4			X						
Sterculiaceae	*Theobroma cacao* L.	Cocoa (cacao)	Cu	t	edi	3.5				X	X				
Strelitziaceae	*Strelitzia reginae* Aiton [1]	(ave del paraíso)	Cu	h	orn	0.2	<0.1			X					X
Tiliaceae	*Triumfetta semitriloba* Jacq.	(pegadillo)	NaQ	h	non	36.8		X	X	X					
Tropaeolaceae	*Tropaeolum majus* L.	Nasturtium (mastuerzo)	Cu	v	med	2.2	0.2		X	X	X		X	X	X
Turneraceae	*Turnera subulata* Sm. [1]		Cu	h	orn	0.2	0.6			X				X	X
Turneraceae	*Turnera ulmifolia* L. [1]		Cu	h	orn	0.7	1.3			X	X			X	X
Ulmaceae	*Trema micrantha* (L.) Blume	(sapan, huevos de paloma)	NaQ	t	non	7.0			X						
Urticaceae	*Pilea depressa* (Sw.) Blume [1]		Cu	h	orn	3.3	2.5			X				X	X
Urticaceae	*Pilea microphylla* (L.) Liebm.		AcQ	h	orn	1.6	0.1		X		X			X	X
Urticaceae	*Pilea nummulariifolia* (Sw.) Wedd. [1]		Cu	h	orn		0.3							X	
Urticaceae	*Pilea serpyllacea* (Kunth) Liebm. [1]		Cu	h	orn	0.5	2.5			X				X	X
Urticaceae	*Pilea* sp. 1 [1]		AcQ	h	orn		<0.1								X
Urticaceae	*Urera baccifera* (L.) Gaudich. ex Wedd. [1]		Cu	sh	med	0.2	<0.1			X				X	
Urticaceae	*Urera caracasana* (Jacq.) Griseb.		NaQ	ssh	non	0.4			X						
Valerianaceae	*Valeriana chaerophylloides* Sm.	(valeriana)	NaQ	v	med	25.6	0.1	X	X	X	X				X
Verbenaceae	*Aloysia triphylla* Royle	(cedrón)	Cu	sh	med	0.2	0.1				X				X
Verbenaceae	*Citharexylum gentryi* Moldenke	(sauco macho, palo de vaca)	Es	t	tim	0.2					X				
Verbenaceae	*Clerodendrum philippinum* Schauer		Es	sh	non	6.2				X	X				
Verbenaceae	*Clerodendrum thomsonae* Balf. [1]	Bleeding Hearts (corazón de Cristo, sangre de Cristo)	Cu	v	orn	0.7	1.0		X	X	X		X	X	X
Verbenaceae	*Clerodendrum* x *speciosum* Dombrain [1]		Cu	v	orn		0.1								X
Verbenaceae	*Duranta repens* ssp. 1 L. [1]	(San Jacinto)	Cu	sh	orn		0.1							X	X
Verbenaceae	*Lantana camara* L.	Curse of India, Koster's Curse (supirrosa)	Es	sh	non	34.6	13.8	X	X	X	X			X	X
Verbenaceae	*Lantana montevidensis* (Spreng.) Briq.		Cu	v	med		0.1							X	X
Verbenaceae	*Phyla nodiflora* var. *reptans* (HBK.) Moldenke		NaQ	h	non	1.5	0.1	X							X
Verbenaceae	*Priva lappulacea* (L.) Pers.	(cadillo)	Ac	h	non	46.9	1.9	X	X	X	X	X	X	X	X
Verbenaceae	*Stachytarpheta cayennensis* (Rich.) M. Vahl	(piche de gato, rabo de zorro)	AcQ	ssh	non	*78.0	0.5	X	X	X	X			X	X
Verbenaceae	*Tectona grandis* L. f.	Teak (teca)	Cu	t	tim	4.9	<0.1		X	X	X			X	
Verbenaceae	*Verbena litoralis* Kunth	(verbena)	NaQ	h	non	57.9	0.1	X	X	X	X				X

Family	Species name	Common name English (local-Spanish)	Introduction status in Galapagos	Growth form	Use	% of visited rural properties	% of fully surveyed urban properties	Floreana_rural	Isabela_rural	San Cristobal_rural	Santa Cruz_rural	Floreana_urban	Isabela_urban	San Cristobal_urban	Santa Cruz_urban
Violaceae	*Viola odorata* L.	Sweet Violet (violeta de jardin)	Cu	h	med	0.4	0.1				X				X
Vitaceae	*Vitis vinifera* L.	Grape (uva)	Cu	v	edi	3.3	1.5		X	X	X		X	X	X
Zingiberaceae	*Alpinia purpurata* (Vieill.) K. Schum. [1]		Cu	h	orn	0.9	0.3			X				X	X
Zingiberaceae	*Alpinia sanderae* Sander [1]		Cu	h	orn	0.9	<0.1		X	X	X				X
Zingiberaceae	*Alpinia zerumbet* (Pers.) B.L. Burtt & R.M. Sm.		Cu	h	orn	2.2	0.1			X	X			X	X
Zingiberaceae	*Costus guanaiensis* var. *tarmicus* (Loes.) Maas [1]	(caña agria)	Cu	h	orn	1.1	0.1		X	X	X			X	
Zingiberaceae	*Etlingera elatior* (Jack) R.M. Sm.		Cu	h	orn	0.2					X				
Zingiberaceae	*Hedychium coronarium* J. König		Es	h	orn	4.8				X	X				
Zingiberaceae	*Zingiber officinale* Roscoe	Ginger (gengibre)	Cu	h	med	0.2	0.2			X				X	X
Zygophyllaceae	*Tribulus cistoides* L.	(cacho de chivo)	NaQ	v	non	0.2	10.8	X				X	X	X	X
Zygophyllaceae	*Tribulus terrestris* L.	(cacho de chivo)	NaQ	v	non		0.3						X		X
