# Supplementary material for: An Extensive Alien Plant Inventory from the Inhabited Areas of Galapagos
Source: PLoS One. 2010 Apr 21;5(4):e10276. doi: 10.1371/journal.pone.0010276 (PMC2858082; doi:10.1371/journal.pone.0010276)
Supplement: Text S1 — Complementary details on data collection. (0.01 MB PDF) [file pone.0010276.s002.pdf]

## Complementary details on data collection

Maps of property boundaries were obtained from the municipality. All new subdivisions were recorded with GPS. We used these maps to ensure all rural properties were surveyed, and to select the random samples of urban properties within town blocks. To select the random samples, we labelled the properties in each block and then used a random number generator to select five of the numbers available. If there were less than five properties in a block we surveyed them all. To ensure we could match properties to digital maps later, we recorded a GPS location within each visited property. Each property was surveyed by either one person or a two-person team. The land owner was informed of the purpose of the study and, in most cases, gave permission to carry out the survey right away. All urban properties were small (less than one hectare), hence it was possible to survey the entire area. In contrast rural properties ranged in size from less than one hectare to over 300 ha and it was rarely possible to cover the whole area. Only specific areas were visited: immediate surroundings of houses, cultivated zones, roads and trails and property's perimeter. Any area reported by the owner as having alien plants was also visited.

Whenever possible, we interviewed the land owner or tenant to collect information on plant uses, local common names, weedy behaviour, date and history of introduction, history of the property's ownership and land use.

The time needed to complete the survey of one property was extremely variable (from 20 minutes to one day) depending on the size of the property, how clear its boundaries were, the total number of species, and number of unknown or new species, the state of the vegetation (many farms were abandoned and overgrown by vegetation), and the presence of unfriendly dogs. On rural properties, it proved helpful to work with a local community member who knew the land owners and the area.

In order to define if a cultivated species had naturalized (escaped), we considered the following factors: how often individuals of that particular species were found "not under cultivation" and how often with fruits; how often seedlings and juveniles from non-cultivated plants were observed; how far from the parent plant(s) these seedlings and juveniles were growing, and if some of these were escaping in adjacent properties; what was their dependence on human care; and the characteristics of the species. The information we gathered revealed gradients along the naturalization continuum. However some factors were difficult to evaluated, for example how much the survival and success of the individuals depended on human intervention (often unintentionally). This is why we included both casual and naturalized in our definition of naturalized species. If a species recorded as cultivated in the Galapagos checklist was found naturalized, its status origin was updated to escaped.
